# Cabozantinib selectively induces proteasomal degradation of p53 somatic mutant Y220C and impedes tumor growth

**DOI:** 10.1016/j.jbc.2025.108167

**Published:** 2025-01-08

**Authors:** Fang Lin Lv, Lu Zhang, Cheng Ji, Lei Peng, Mingxian Zhu, Shumin Yang, Shunli Dong, Mingxuan Zhou, Fanfan Guo, Zhenyun Li, Fang Wang, Youguo Chen, Jinhua Zhou, Xingcong Ren, Genhai Shen, Jin-Ming Yang, Bin Li, Yi Zhang

**Affiliations:** 1 Department of Hepatopancreatobiliary Surgery, Suzhou Ninth Hospital Affiliated to Soochow University, Suzhou, Jiangsu, China; 2 Department of Pharmacology, College of Pharmaceutical Sciences, Soochow University, Suzhou, Jiangsu, China; 3 Department of Respiratory Medicine, First Affiliated Hospital, Soochow University, Suzhou, Jiangsu, China; 4 Department of Gynecology and Obstetrics, First Affiliated Hospital, Soochow University, Suzhou, Jiangsu, China; 5 Department of Cancer Biology and Toxicology, Markey Cancer Center, University of Kentucky, College of Medicine, Lexington, Kentucky, USA

**Keywords:** cabozantinib, p53Y220C, proteasomal degradation, tumor cells, USP7

## Abstract

Inactivation of p53 by mutations commonly occurs in human cancer. The mutated p53 proteins may escape proteolytic degradation and exhibit high expression in tumors and acquire gain-of-function activity that promotes tumor progression and chemo-resistance. Therefore, selectively targeting of the gain-of-function p53 mutants may serve as a promising therapeutic strategy for cancer prevention and treatment. In this study, we identified cabozantinib, a multikinase inhibitor currently used in the clinical treatment of several types of cancer, as a selective inducer of proteasomal degradation of the p53-Y220C mutant. We demonstrate that cabozantinib disrupts the interaction between p53Y220C and USP7, a deubiquitylating enzyme, resulting in the dissociation of p53Y220C protein from its binding with USP7 and subsequent ubiquitination and degradation mediated by CHIP (the carboxyl terminal of Hsp70-interacting protein). We also show that cabozantinib displays preferential cytotoxicity to p53Y220C-harboring cancer cells both *in vitro* and *in vivo*. This study demonstrates a novel, p53-Y220C mutant–targeted anticancer action and mechanism for cabozantinib and provides the rationale for use of this drug in the treatment of cancers that carry the p53-Y220C mutation.

p53 inactivation commonly occurs in human cancers due to gene deletion or mutations, resulting in uncontrolled cell proliferation, increased genomic instability, and cancer progression (1). TP53 mutations in cancer usually lead to loss of the WT-like activity of p53 and exhibit dominant negative effects on remaining WT p53, conferring oncogenic function (2). These gain-of-function (GOF) oncogenic p53 mutant proteins may modulate the function of other transcriptional regulators (3) or upregulate chromatin regulatory genes (4), contributing to apoptosis inhibition, therapy resistance, invasion and metastasis, and immune evasion (5).

Currently, the majority of small molecules that target p53 mutations are broad-spectrum p53 restorers, and all of them are insufficient to fully restore the WT function of p53 (6, 7). Therefore, targeted therapeutic strategies against specific mutant types of p53 may be a promising approach for exploring and testing. P53^Y220C^ is the most prevalent mutation in certain tumor types (8, 9) and ranked as the sixth most frequent *TP53* mutation across 12 tumor types in The Cancer Genome Atlas Pan-Cancer study (10). TP53-Y220C is the first type of mutation observed in ovarian serous carcinoma at a similar frequency to TP53-R273H (11) and is prevalent in head and neck squamous cell carcinoma and is a relative hotspot mutation in oropharyngeal squamous cell carcinoma (12). This mutation has also been identified in hepatocellular carcinoma (1% frequency), gastric cancer (1% frequency), pancreatic cancer (1% frequency), and esophageal carcinoma (0.7% frequency) (13). Several clues suggest that p53-Y220C mutation exhibits GOF features. For instance, p53-Y220C mutant physically interacts with multiple isoforms of p63 and p73, potentially promoting tumor progression (14). Moreover, over 80% p53-Y220C mutant protein rapidly unfolds at physiological temperature, effectively disrupting p53 signaling and driving tumorigenesis (15). Upregulation of stathmin, a microtubule destabilizer, by the p53-Y220C mutant contributes to chemotherapy resistance (16). Furthermore, this mutant affects cytochrome c oxidase 2 and copper transporter 1, thereby influencing intracellular copper homeostasis in hepatoma carcinoma cells (17). Structure analysis of p53-Y220C protein reveals a solvent-accessible cleft in the core domain of the druggable interface (18). Although the therapeutic strategy targeting restoration of function for p53-Y220C have been explored (19), use of small molecules to destruct p53^Y220C^ has not been documented.

Prompted by our serendipitous observation that tumors harboring different p53 mutants exhibited distinct responses to the combination treatment of cabozantinib and EGFR inhibitor in NSCLC patients with EGFR mutations, we conducted analyses to investigate the effects of cabozantinib on p53 expression in a panel of human tumor cell lines harboring either mutant or WT p53. Interestingly, we found that cabozantinib, a multityrosine kinase inhibitor, can disrupt the interaction between USP7 and p53^Y220C^ proteins and selectively degrade p53^Y220C^ protein through the CHIP-mediated ubiquitin-proteasomal pathway. Further, we showed both *in vitro* and *in vivo* that cabozantinib exhibit preferential cytotoxicity against cancer cells harboring p53-Y220C mutation. Therefore, targeting of p53^Y220C^ protein degradation using cabozantinib may offer a promising therapeutic strategy for the treatment of cancers expressing this mutant variant.

## Results

### Cabozantinib elicits selective downregulation of p53^Y220C^

**Figure 1:**
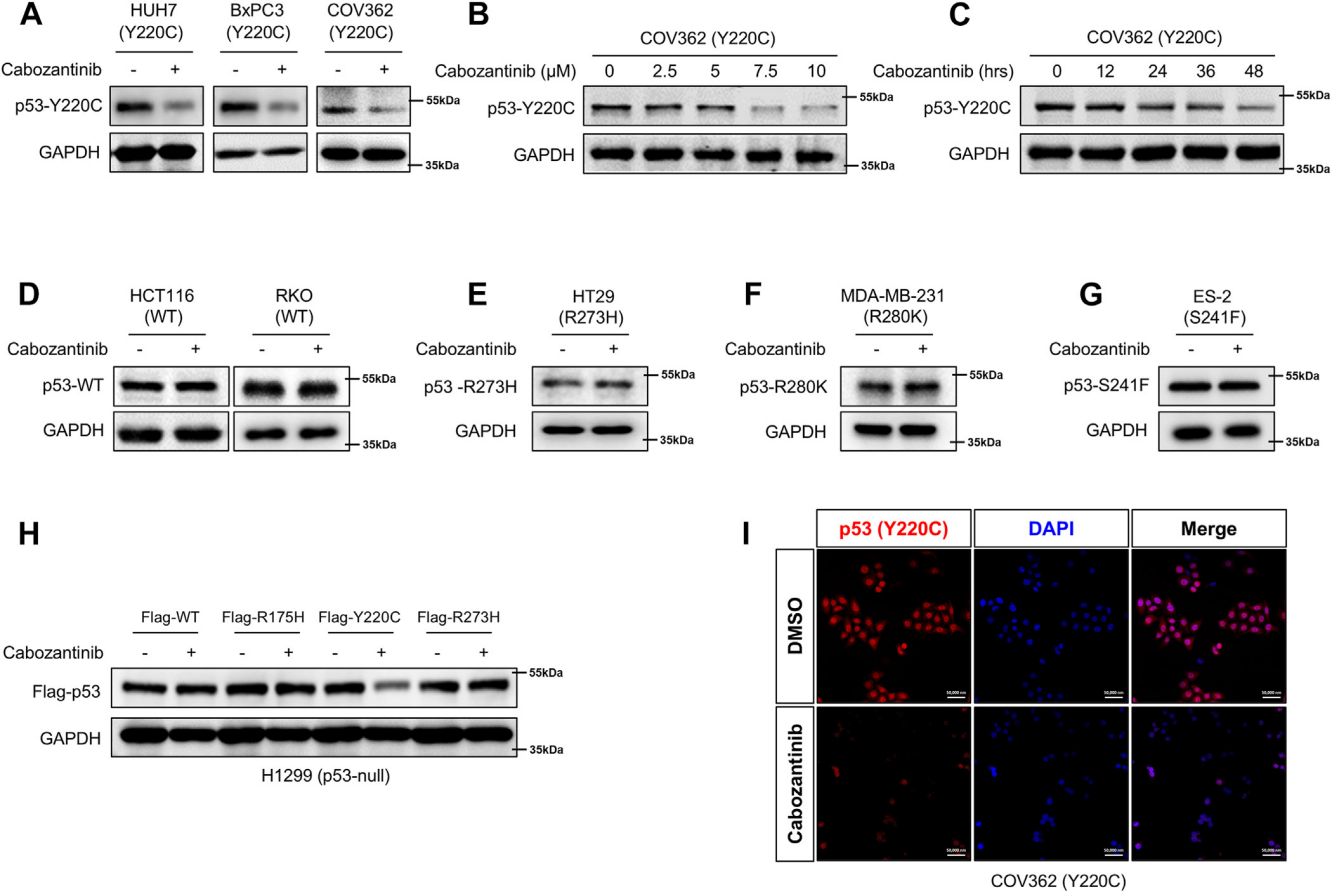
**Cabozantinib elicits selective downregulation of p53-Y220C.***A*, Immunoblotting for p53 in HUH7, BxPC3, and COV362 cells treated with 10 μM cabozantinib for 48 h. GAPDH was used as a loading control. *B*, COV362 cells were treated with the indicated concentrations of cabozantinib for 48 h. At the end of treatment, the level of p53 was examined by immunoblotting. GAPDH was used as a loading control. *C*, COV362 cells were treated with 10 μM cabozantinib and harvested at the indicated time points, followed by immunoblotting analysis to determine p53 expression. GAPDH was used as a loading control. *D–G*, Immunoblotting for p53 in (D) HCT116, RKO cells (p53^WT^), (*E*) HT29 (p53^R273H^), (*F*) MDA-MB-231 (p53^R280K^), (*G*) ES-2 (p53^S241F^) treated with 10 μM cabozantinib for 48 h. GAPDH was used as a loading control. *H*, H1299 (p53^null^) cells were transfected with Flag-p53^WT^, Flag-p53^R175H^, Flag-p53^Y220C^, or Flag-p53^R273H^ plasmids for 24 h followed by treatment with 10 μM cabozantinib for 48 h. Flag protein was determined by immunoblotting. GAPDH was used as a loading control. *I*, Immunofluorescence for p53^Y220C^ using COV362 cells treated with DMSO or cabozantinib (10 μM) for 48 h. The scale bar represents 50 μm.

To investigate the effect of cabozantinib on p53^Y220C^ expression, we treated a panel of human tumor cell lines with different p53 mutants or WT p53 with cabozantinib and then examined the amount of p53 protein using immunoblotting. As shown in Fig. 1*A*, cabozantinib caused an evident reduction in the level of p53^Y220C^ protein in HUH7 (liver cancer), BxPC3 (pancreatic cancer), and COV362 (ovarian cancer) cell lines. The effect of cabozantinib on p53^Y220C^ protein downregulation exhibited a dose-dependent and time-dependent manner (Fig. 1, *B* and *C*). By contrast, cabozantinib treatment barely had any effect on the levels of p53^WT^ (Fig. 1*D*) or the other GOF mutant p53 (R273H, R280K, and S241F) (Fig. 1, *E–G*). Similar results were obtained in H1299 cells (p53-null) expressing exogenous p53^WT^, p53^R175H^, p53^Y220C^, or p53^R273H^ (Fig. 1*H*). The decline in total p53^Y220C^ protein level following cabozantinib treatment was confirmed by immunofluorescence staining in COV362 cells (Fig. 1*I*). Cabozantinib does not appear to affect the transcription of p53^Y220C^, as the expression of p53^Y220C^ mRNA was not changed in HUH7, BxPC3, and COV362 cells treated with cabozantinib (Fig. S1*A*). Cabozantinib, a small-molecule tyrosine kinase inhibitor against multiple targets including c-MET, VEGFR2, and AXL (20). To determine whether the effect of cabozantinib on p53-Y220C protein involves these targets, we employed siRNA to silence c-MET, VEGFR2, or AXL in COV362, BxPC3, or HUH7 cells. We showed that knockdown of c-MET, VEGFR2, or AXL did not affect the levels of p53-Y220C mutant protein (Fig. S1, *B–J*), suggesting no involvement of those kinase in the cabozantinib-induced degradation of TP53(Y220C) protein.

### Cabozantinib causes the lysine K373-mediated ubiquitin-proteasomal degradation of p53Y220C protein

**Figure 2:**
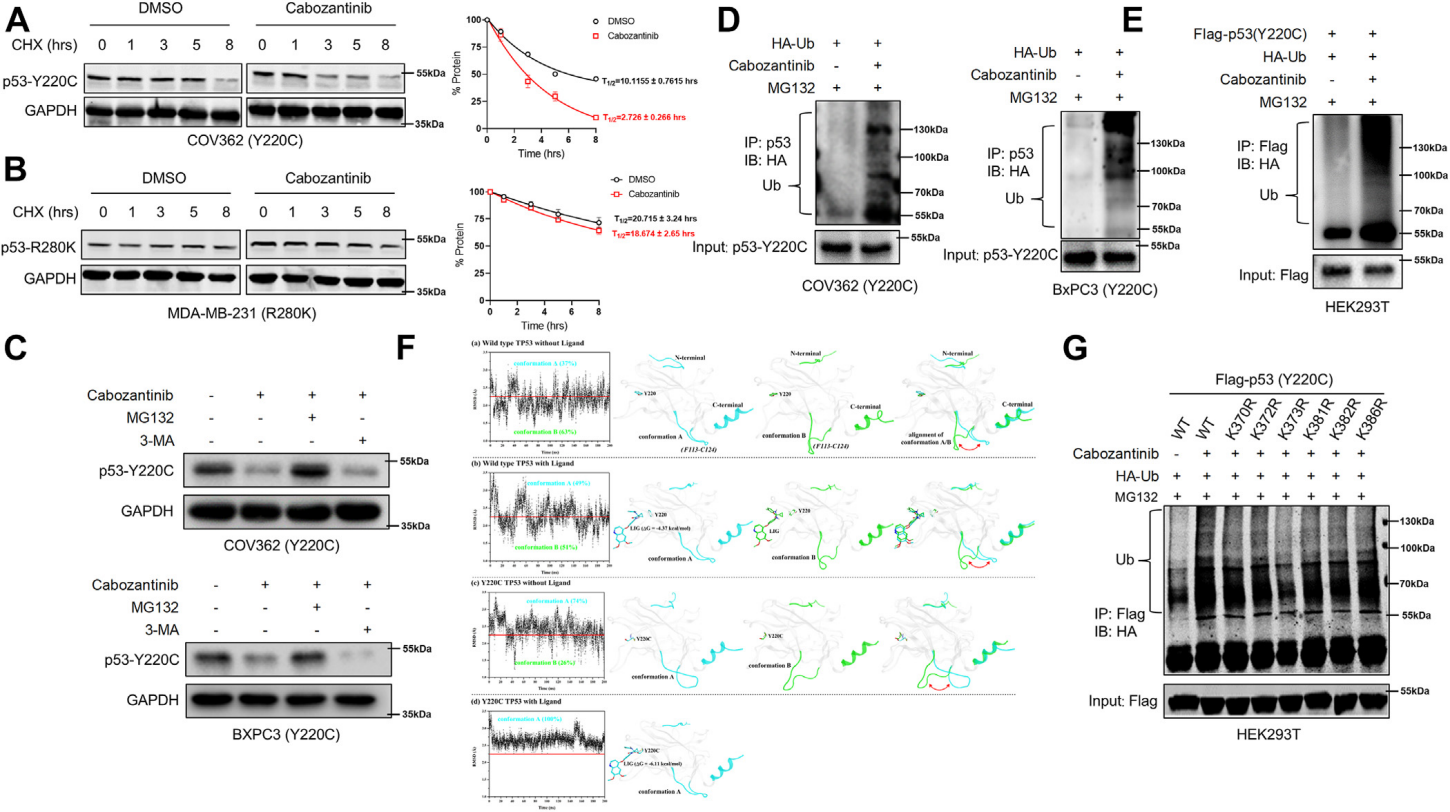
**Cabozantinib causes proteasomal-mediated turnover of p53-Y220C at lysine K373.***A* and *B*, Immunoblotting for p53 in COV362 cells (*A*) and MDA-MB-231 cells (*B*) were treated with DMSO or 10 μM cabozantinib for 48 h and then treated with 50 μg/ml cycloheximide (CHX) at different time points time before harvesting the cell for immunoblotting. Bars are mean ± SD (n = 3). *C*, Immunoblotting for p53 in COV362 and BxPC3 cells treated with 10 μM cabozantinib for 48 h, either in the presence or absence of MG132 (10 μM) or 3-MA (2 mM) for 8 h before harvesting the cells. GAPDH was used as a loading control. *D*, COV362 and BxPC3 cells transfected with HA-Ub were treated with DMSO or 10 μM cabozantinib for 48 h and then 10 μM MG132 was added 8 h before harvesting the cells. Cell lysates were immunoprecipitated with an anti-p53 antibody. The immunoprecipitates and input were probed for HA and p53 by immunoblotting. *E*, HEK293T cells transfected with HA-Ub and Flag-p53^Y220C^ were treated with DMSO or 10 μM cabozantinib for 48 h and then 10 μM MG132 was added 8 h before harvesting the cells. Cell lysates were immunoprecipitated with an anti-Flag antibody. The immunoprecipitates and input were probed for HA and Flag by immunoblotting. *F*, Cluster analysis results of (a) free WT TP53 model, (b) WT TP53-ligand complex model, (c) free Y220C TP53 model, and (d) Y220C TP53-ligand complex model. *G*, HEK293T cells were transfected with HA-Ub and site-directed mutagenesis (Flag-p53^Y220C/WT^, Flag-p53^Y220C/K370R^, Flag-p53^Y220C/K372R^, Flag-p53^Y220C/K373R^, Flag-p53^Y220C/K381R^, Flag-p53^Y220C/K382R^, Flag-p53^Y220C/K386R^) were treated with 10 μM cabozantinib for 48 h and then10 μM MG132 was added 8 h before harvesting the cells. Cell lysates were immunoprecipitated with an anti-Flag antibody. The immunoprecipitates and input were probed for HA and Flag by immunoblotting.

Next, we determined whether cabozantinib has any effects on the turnover of p53^Y220C^ protein. Cycloheximide-chase analysis revealed a significant reduction in the half-life of p53^Y220C^ in COV362, BxPC3, and HUH7 cell lines treated with cabozantinib (Fig. 2*A* and Fig. S2*A*). The half-life of p53^R280K^ (MDA-MB-231 cell line) and p53^R273H^ (HT29 cell line) proteins was not affected by cabozantinib treatment (Fig. 2*B* and Fig. S2*B*). MG132, a proteasomal inhibitor, could block the cabozantinib-induced turnover of the p53^Y220C^ protein, but the autophagy inhibitor 3-methyladenine could not (Fig. 2*C*), suggesting that cabozantinib promotes the degradation of p53^Y220C^ *via* proteasomal degradation. Furthermore, enhanced ubiquitination of p53^Y220C^ protein was observed following cabozantinib treatment (Fig. 2*D*). Similar results were obtained in HEK293T cells expressing ectopic p53^Y220C^ (Fig. 2*E*). Enhanced endogenous ubiquitination of p53Y220C was observed upon cabozantinib treatment (Fig. S2, *C* and *D*). These results indicate that the degradation of p53^Y220C^ protein induced by cabozantinib is mediated through the ubiquitin-proteasomal pathway.

Based on our findings that cabozantinib induced selective degradation of p53^Y220C^ but did not affect other mutant p53 variants and that the presence of a hydrophobic pocket on the surface of p53^Y220C^ protein (21), we wanted to know whether cabozantinib could interact with the hydrophobic pockets on the surface of p53^Y220C^ and subsequently cause structural alterations and promote ubiquitination and degradation of p53^Y220C^ protein. We performed molecular docking experiments, and the binding mode between TP53 protein and cabozantinib was shown in Fig. S2*E*. Based on the molecular dynamic simulations, we performed the cluster analysis and proposed a model of TP53–cabozantinib complex (Fig. 2*F*). In the free WT TP53 model (Fig. 2*F* (a)), TP53 protein tended to present two different conformations: one conformation, referred to as conformation A, involves the closure of the F113-C124 loop towards the C-terminal region of TP53 protein; another conformation, referred to as conformation B, this loop was positioned away from the C-terminal region, suggesting that when cabozantinib did not bind with TP53 protein, there was a greater tendency for the loop to adopt conformation B (Fig. 2*F* (a)). Interestingly, in the WT TP53–cabozantinib complex model and the free Y220C TP53 model (Fig. 2*F* (b)-(c)), the loop exhibits a greater inclination towards conformation A, although both display conformation A and conformation B, suggesting that both of the cabozantinib and Y220C mutation can cause a preference for conformation A in the TP53 protein loop, with the Y220C mutation exerting a stronger influence on this trend (Fig. 2*F* (c)). In the Y220C TP53–cabozantinib complex model where both cabozantinib and the Y220C mutation were considered, only conformation A was observed while no evidence of conformation B, further supporting a positive correlation between conformation A and cabozantinib/Y220C mutation. These results suggest that cabozantinib has a more pronounced impact in altering the structure of mutant p53^Y220C^, particularly evident in changes within its C-terminal region, indicating that the Y220C mutation enhances binding affinity between cabozantinib and TP53 protein. Notably, polyubiquitination modification sites responsible for p53 protein degradation are predominantly located at its C-terminus (22) (Fig. S2*F*). To identify the lysine residues within the C-terminal region that are potentially subjected to ubiquitination upon cabozantinib treatment, we used site-directed mutagenesis to generate lysine-to-arginine mutants. Our experiments showed that lysine K373 had an indispensable role in facilitating p53^Y220C^ ubiquitination (Fig. 2*G*), suggesting that lysine K373 of p53^Y220C^ is a crucial mediator of the cabozantinib-induced ubiquitin-proteasomal degradation of p53^Y220C^ protein.

### Cabozantinib induces the CHIP-mediated degradation of p53Y220C by preventing its interaction with USP7

**Figure 3:**
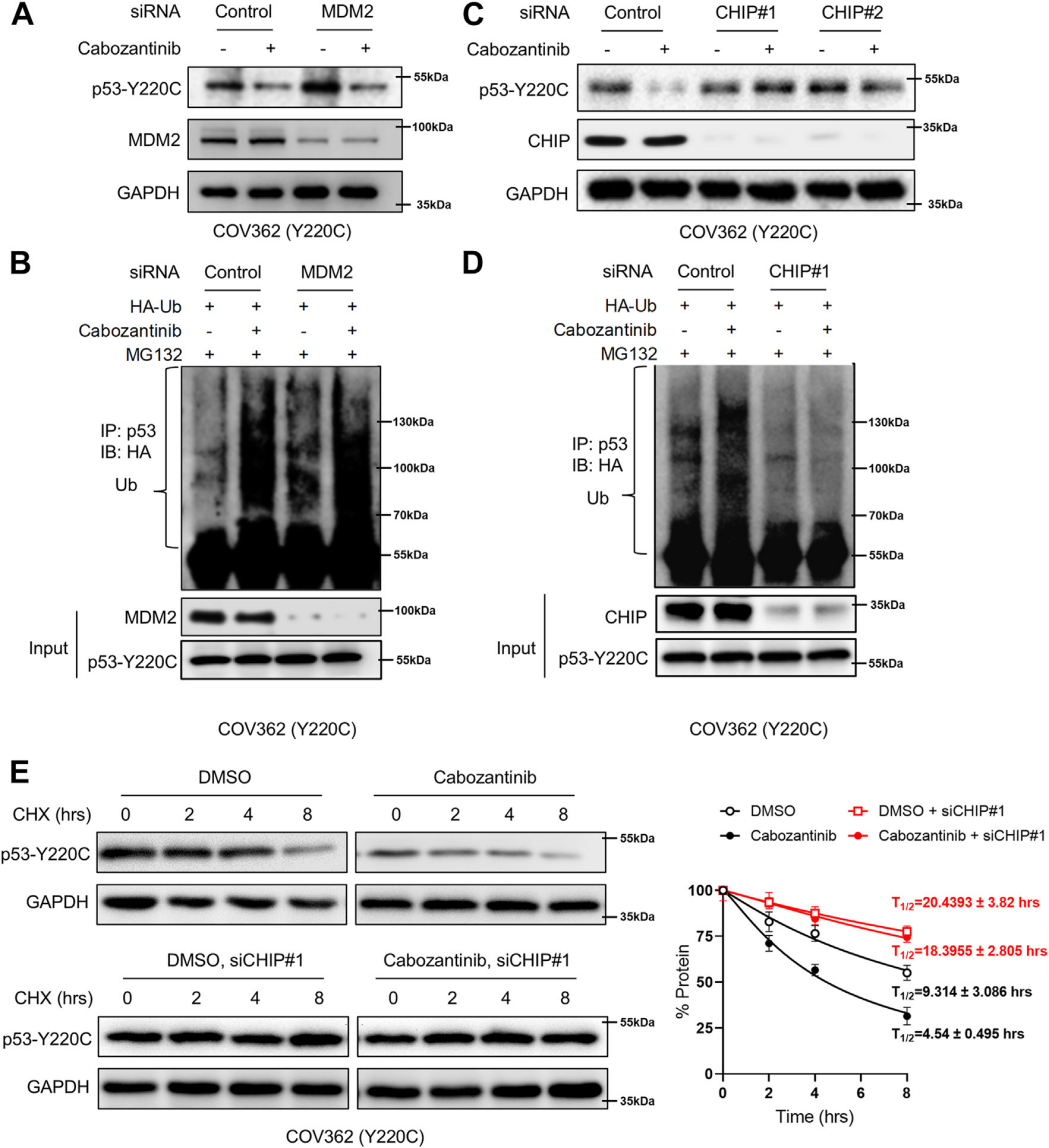
**Cabozantinib induces CHIP-mediated degradation of p53**^**Y220C**^. *A*, COV362 cells were treated with 10 μM cabozantinib for 48 h after transfected with the indicated siRNA for 24 h. P53 and MDM2 were examined by immunoblotting. GAPDH was used as a loading control. *B*, COV362 cells were treated with 10 μM cabozantinib for 400 h after transfected with the indicated siRNA for 24 h and 10 μM MG132 was added 8 h before harvesting the cells. Cell lysates were immunoprecipitated with an anti-p53 antibody. The immunoprecipitates and input were probed for HA and p53 by immunoblotting. *C*, COV362 cells were treated with 10 μM cabozantinib for 48 h after transfected with the indicated siRNA for 24 h. P53 and CHIP were examined by immunoblotting. GAPDH was used as a loading control. *D*, COV362 cells were treated with 10 μM cabozantinib for 48 h after transfected with the indicated siRNA for 24 h and 10 μM MG132 was added 8 h before harvesting the cells. Cell lysates were immunoprecipitated with an anti-p53 antibody. The immunoprecipitates and input were probed for HA and p53 by immunoblotting. *E*, COV362 cells transfected with indicated siRNA for 24 h were treated with DMSO or 10 μM cabozantinib for 48 h and then treated with 50 μg/ml cycloheximide (CHX) at different time points before harvesting the cell for immunoblotting. GAPDH was used as a loading control. Bars are mean ± SD (n = 3).

To gain molecular insight into the proteasomal degradation of p53^Y220C^ protein induced by cabozantinib, we tested several ubiquitin ligases responsible for p53 degradation. Our experiments showed that knockdown of MDM2, a major ubiquitin ligase for p53, did not reverse the cabozantinib-induced p53^Y220C^ degradation in COV362 and BxPC3 cells (Fig. 3, *A* and *B* and Fig. S3, *A* and *B*). Nevertheless, the cabozantinib-induced degradation and ubiquitination of p53^Y220C^ was apparently abolished by knockdown of another ubiquitin ligase for p53, CHIP (Fig. 3, *C* and *D* and Fig. S3, *C* and *D*). In addition, CHIP knockdown resulted in an increased half-life of p53^Y220C^ protein even in the presence of cabozantinib (Fig. 3*E*). These results indicate that CHIP plays a critical role in the cabozantinib-induced ubiquitin-proteasomal degradation of p53^Y220C^ protein.

**Figure 4:**
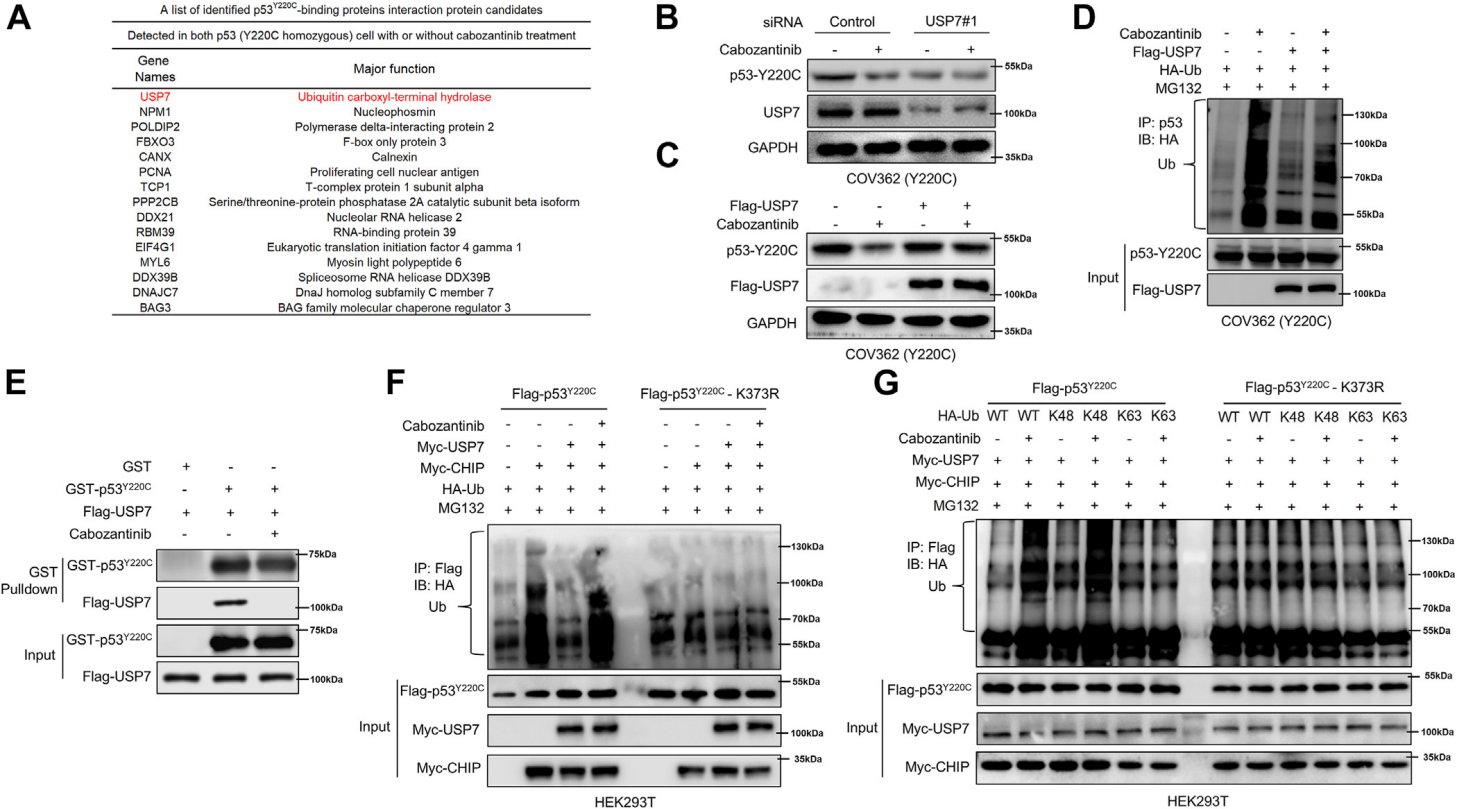
**Cabozantinib disrupts the interaction between USP7 and p53**^**Y220C**^**and induces CHIP-mediated degradation through K48-linked ubiquitylation.***A*, List of the downregulated p53^Y220C^-binding proteins in COV362 cells transfected with either Flag-vector or Flag-p53^Y220C^ upon cabozantinib treatment, as identified by LC-MS/MS. *B*, COV362 cells were treated with 10 μM cabozantinib for 48 h after transfected with the indicated siRNA for 24 h. P53 and USP7 were examined by immunoblotting. GAPDH was used as a loading control. *C*, COV362 cells were treated with 10 μM cabozantinib for 48 h after transfected with the indicated plasmids. P53 and Flag-USP7 were examined by immunoblotting. GAPDH was used as a loading control. *D*, COV362 cells were treated with 10 μM cabozantinib for 48 h after transfected with the indicated plasmids for 24 h and then 10 μM MG132 was added 8 h before harvesting the cells. Cell lysates were immunoprecipitated with an anti-p53 antibody. The immunoprecipitates and input were probed for HA and p53 by immunoblotting. *E*, *In vitro* binding assay: Purified USP7 was incubated with GST-tagged p53 and treated with cabozantinib (10 μM) or DMSO for 24 h, followed by GST pulldown assay and immunoblotting with anti-USP7 or anti-p53 antibody. *F*, HEK293T cells were treated with 10 μM cabozantinib for 48 h after transfected with the indicated plasmids for 24 h and then 10 μM MG132 was added 8 h before harvesting the cells. Cell lysates were immunoprecipitated with an anti-Flag antibody. The immunoprecipitates and input were probed for HA and Flag by immunoblotting. *G*, HEK293T cells were treated with 10 μM cabozantinib for 48 h after transfected with the indicated plasmids for 24 h and then 10 μM MG132 was added 8 h before harvesting the cells. Cell lysates were immunoprecipitated with an anti-Flag antibody. The immunoprecipitates and input were probed for HA and Flag by immunoblotting.

To further determine the mechanism by which cabozantinib induces degradation of p53^Y220C^ *via* CHIP, using the COV362 cells transfected with either Flag-vector or Flag-p53^Y220C^-vector, we performed immunoprecipitation with an anti-Flag antibody, followed by LC-MS/MS analysis to identify proteins that associate with p53^Y220C^ in the presence or absence of cabozantinib. Cabozantinib treatment resulted in altered abundance of several potential p53^Y220C^-binding proteins including USP7, a deubiquitynating enzyme (Fig. 4*A*). Thus, we examined the role of USP7 in the cabozantinib-induced p53^Y220C^ degradation. We showed that knockdown of USP7 in COV362 and BxPC3 cells resulted in significant reduction of p53^Y220C^ protein level, and co-treatment with MG132 rescued the downregulation of p53^Y220C^ upon USP7 knockdown (Fig. S3, *E* and *F*). Further, knockdown of USP7 enhanced ubiquitination of p53^Y220C^ (Fig. S4, *A* and *B*) and prevented further reduction of p53^Y220C^ protein caused by cabozantinib (Fig. 4*B*). Additionally, cabozantinib treatment did not affect USP7 protein level in COV362 and BxPC3 cells harboring p53^Y220C^ (Fig. S4*C*). Subsequently, we performed the cycloheximide chase assay in COV362 (p53^Y220C^) and MCF-7 (p53^WT^) cells with USP7 knockdown. The results of these experiments showed that in the presence or absence of cabozantinib, USP7 knockdown could reduce the half-life of p53^Y220C^ protein, but had no effect on p53^WT^ protein, suggesting that silencing USP7 in p53^Y220C^ cells can produce a similar effect to cabozantinib (Fig. S4, *D* and *E*). Overexpression of USP7 significantly prolonged the half-life of p53^Y220C^ protein (Fig. S5*A*) and effectively counteracted the degradation and ubiquitination of p53^Y220C^ induced by cabozantinib (Fig. 4, *C* and *D*).

To demonstrate the effect of cabozantinib on the interaction between USP7 and p53^Y220C^, we conducted GST-pulldown and co-immunoprecipitation assays in the cells treated with cabozantinib. Our *in vitro*–binding assays revealed that cabozantinib directly disrupted the binding between p53 and USP7(Fig. 4*E*). Also, we showed a decrease in both exogenous USP7–p53^Y220C^ interaction in HEK293T cells and endogenous USP7-p53^Y220C^ interaction in COV362 cells following treatment with cabozantinib (Fig. S5, *B* and *C*). Moreover, we showed that CHIP knockdown abrogated the downregulation of p53^Y220C^ caused by USP7 knockdown (Fig. S5*D*), and simultaneous knockdown of CHIP and USP7 attenuated the enhanced ubiquitination of p53^Y220C^ in the cells subjected to USP7 knockdown (Fig. S5*E*). These results suggest that USP7 knockdown elicited similar effects to cabozantinib on p53^Y220C^ and validated the crucial role of USP7 in the cabozantinib-induced, CHIP-mediated degradation of p53^Y220C^ protein. Cabozantinib treatment enhanced the ubiquitination of p53^Y220C^ in the cells cotransfected with USP7, CHIP, and Ub plasmids (compare lane 3 with lane 4, Fig. 4*F*), but this effect was not observed in the cells harboring p53^Y220C/K373R^ mutant (compare lane 7 with lane 8, Fig. 4*F*). USP7 can effectively remove K48- and K63-linked ubiquitin chains (23); thus, we examined the effect of USP7 on different types of p53^Y220C^ ubiquitination using the plasmids encoding WT or various lysine-mutant ubiquitin. We observed that USP7 efficiently attenuated CHIP-mediated p53^Y220C^ K48-linked ubiquitination (Fig. S5, *F* and *G*). Notably, cabozantinib treatment increased the K48-linked p53^Y220C^ ubiquitination as compared to that observed in the p53^Y220C/K373R^ mutant (Fig. 4*G*). Collectively, these results demonstrate that cabozantinib can disrupt the interaction between USP7 and p53^Y220C^, inducing the CHIP-mediated degradation through K48-linked ubiquitination.

### Cabozantinib preferentially impedes p53^Y220C^-harboring tumor growth through the degradation of p53^Y220C^ both *in vitro* and *in vivo*

**Figure 5:**
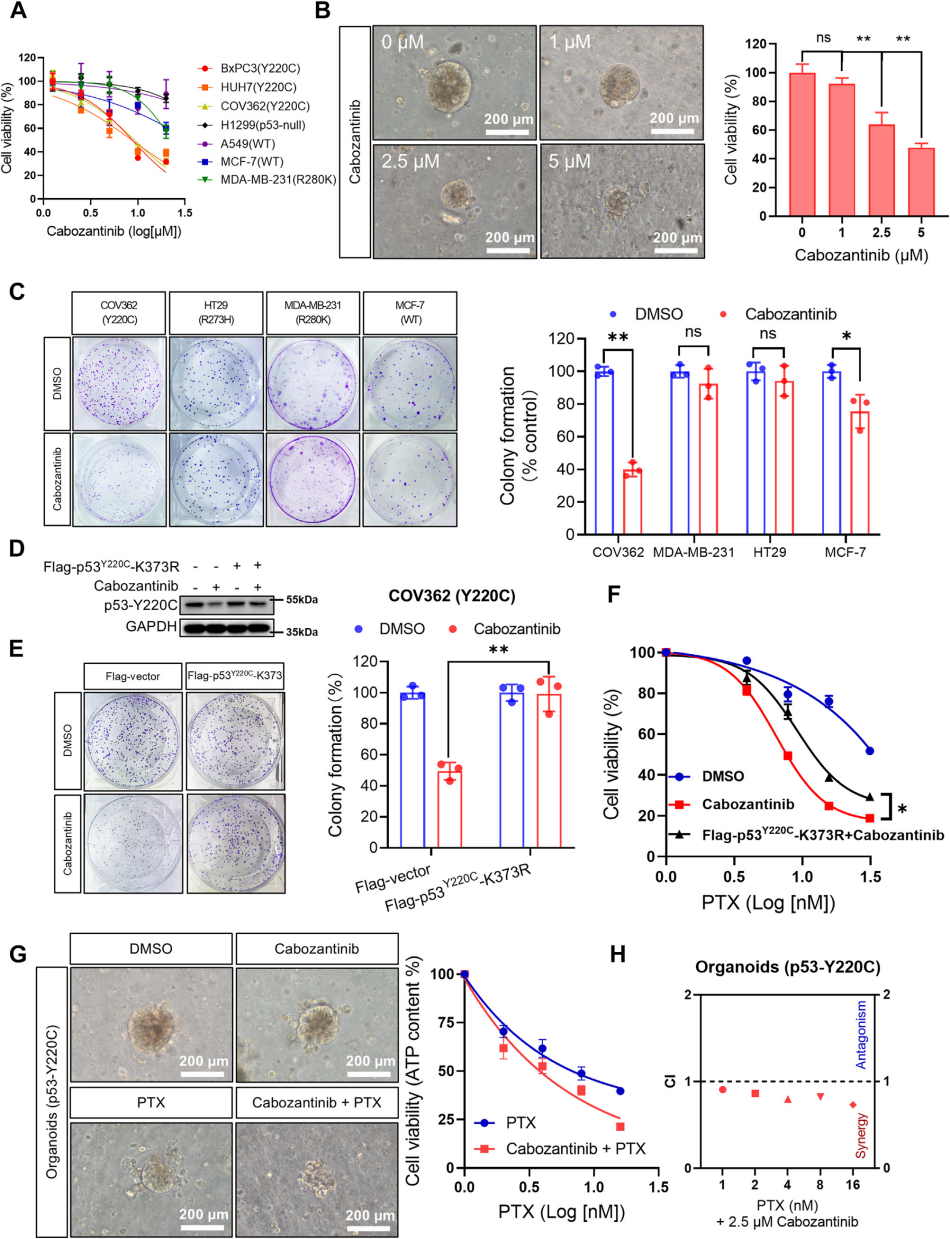
**Cabozantinib’s preferential cytotoxic effect on p53**^**Y220C**^**-harboring tumor cells growth through the degradation of p53**^**Y220C**^***in vitro*.***A*, Cell viability assay for the indicated cell lines upon cabozantinib treatment for 48 h. *B*, Cell viability assay for the non-small cell lung cancer organoids (p53^Y220C^) treated with the indicated concentration of cabozantinib for 48 h. Representative images (*left*) and a summarized graph (*right*). Data shown are the mean ± SD (n = 3). *C*, Colony formation assays for the indicated cell lines upon 10 μM cabozantinib treatment for 14 days. Data shown are the mean ± SD (n = 3). *D–F*, (*D*) Flag-vector or Flag-p53^Y220C^-K373R COV362 cells were treated with or without cabozantinib for 48 h. P53 protein was examined by immunoblotting. GAPDH was used as a loading control. *E*, Colony formation assays for Flag-vector or Flag-p53^Y220C^-K373R COV362 cells upon 10 μM cabozantinib treatment for 14 days. Data shown are the mean ± SD (n = 3). *F*, Cell viability assay for the COV362 cells were treated with the indicated concentrations of paclitaxel for 48 h in the presence or absence of cabozantinib (10 μM) or Flag-p53^Y220C^-K373R plasmid transfection. Data shown are the mean ± SD (n = 3). *G*, Cell viability assay for the non-small cell lung cancer organoids (p53^Y220C^) treated with DMSO, cabozantinib (2.5 μM), paclitaxel (8 nM), or cabozantinib (2.5 μM) plus paclitaxel (1, 2, 4, 8, 16 nM) for 48 h. *Left*: Representative images; *right*: a summary graph. Data shown are the mean ± SD (n = 3). *H*, combination index (CI) values. CI < 1, synergism; CI > 1, Antagonism.

Since cabozantinib can cause selective downregulation of p53^Y220C^, we queried whether the p53^Y220C^-harboring tumor cells are more sensitive to the cytotoxicity of this drug. We compared the cabozantinib sensitivity in cancer cell lines possessing different p53 status and found that cabozantinib was significantly more efficacious in reducing the cellular viability of BxPC3, HUH7, and COV362 cell lines harboring the p53^Y220C^ than that of H1299 (p53^null^), A549 (p53^WT^), MCF-7 (p53^WT^), or MDA-MB-231 (p53^R280K^) cells (Fig. 5*A* and Table S1). We also determined the effect of cabozantinib on apoptosis and autophagy and showed demonstrating that 10 μM cabozantinib did not induce apoptosis but elicited minimal autophagy (Fig. S6, *A* and *B*). Higher sensitivity of tumor cells harboring p53^Y220C^ mutation to cabozantinib was also observed in the tumor organoids derived from the patient specimens of lung cancer with detectable TP53^Y220C^ (Fig. 5*B*). Cabozantinib showed strong inhibitory effect on colony formation of COV362 cells harboring the p53^Y220C^, but minimal effect on HT29 (p53^R273H^), MDA-MB-231 (p53^R280K^), and MCF-7 (p53^WT^) cells (Fig. 5*C*). Considering that knockdown USP7 in p53^Y220C^ cells elicits a comparable effect to the degradation of p53^Y220C^ protein triggered by cabozantinib, we determined the effect of USP7 silencing on cellular growth. Our results demonstrated that knockdown of USP7 alone yielded a similar effect to treatment with cabozantinib. Furthermore, simultaneous silencing of USP7 and treatment with cabozantinib did not exhibit further inhibitory effect on cellular growth (Fig. S6, *C* and *D*). Notably, overexpression of USP7 substantially rescued the cellular viability and tumor growth in the presence of cabozantinib (Fig. S6, *E* and *F*). To demonstrate that the stronger antitumor effect of cabozantinib on tumor cells harboring p53^Y220C^ mutant is mediated by its role in promoting p53^Y220C^ protein degradation, we tested the COV362 and BxPC3 cells with ectopic expression of p53^Y220C/K373R^. The results showed that cabozantinib sensitivity of P53-Y220C cells subjected to K373R mutation was significantly decreased (Fig. S6, *G* and *H*). Furthermore, the ectopic expression of this mutant gene rescued the cabozantinib-mediated degradation of p53-Y220C (Fig. 5*D* and Fig. S6*I* (a)). Also, we showed that the cells with ectopic expression of p53^Y220C/K373R^ were less sensitive to cytotoxic effect of cabozantinib than the cells without ectopic expression of p53^Y220C/K373R^ (Fig. 5*E* and Fig. S6*I* (b)-(c)), indicating that expression of this mutant p53 can abrogate the effect of the cytotoxic effect of cabozantinib on the mutant-harboring tumor cells.

It was reported that p53^Y220C^ has a role in upregulating stathmin expression and conferring tumor cell resistance to cytotoxic agents such as paclitaxel (PTX) and vinblastine (16). Therefore, we tested whether pharmacological degradation of p53^Y220C^ by cabozantinib could sensitize the tumor cells harboring p53^Y220C^ mutation to PTX. As shown in Fig. 5*F*, the combination of PTX with cabozantinib significantly increased cytotoxicity as compared to PTX alone (IC50 = 8.021 ± 1.259 nM *v.s*. IC50 = 26.725 ± 0.935 nM), but the cells with ectopic expression of p53^Y220C/K373R^ were less sensitive to cytotoxic effect of PTX than the cells without ectopic expression of p53^Y220C/K373R^ (IC50 = 9.126 ± 2.401 nM *v.s*. IC50 = 6.518 ± 2.608 nM). Similar result was obtained in BxPC3 cell line (Fig. S6*J*). Furthermore, we observed that combined treatment of cabozantinib (2.5 μM) with PTX (1, 2, 4, 8, and 16 nM) significantly enhanced cytotoxicity against TP53^Y220C^ organoids compared to either cabozantinib or PTX treatment alone (Fig. 5*G*). Compusyn analysis unveiled that the combination of PTX with cabozantinib were synergistic in inhibiting tumor cell proliferation of the TP53^Y220C^ organoids (Fig. 5*H*).

**Figure 6:**
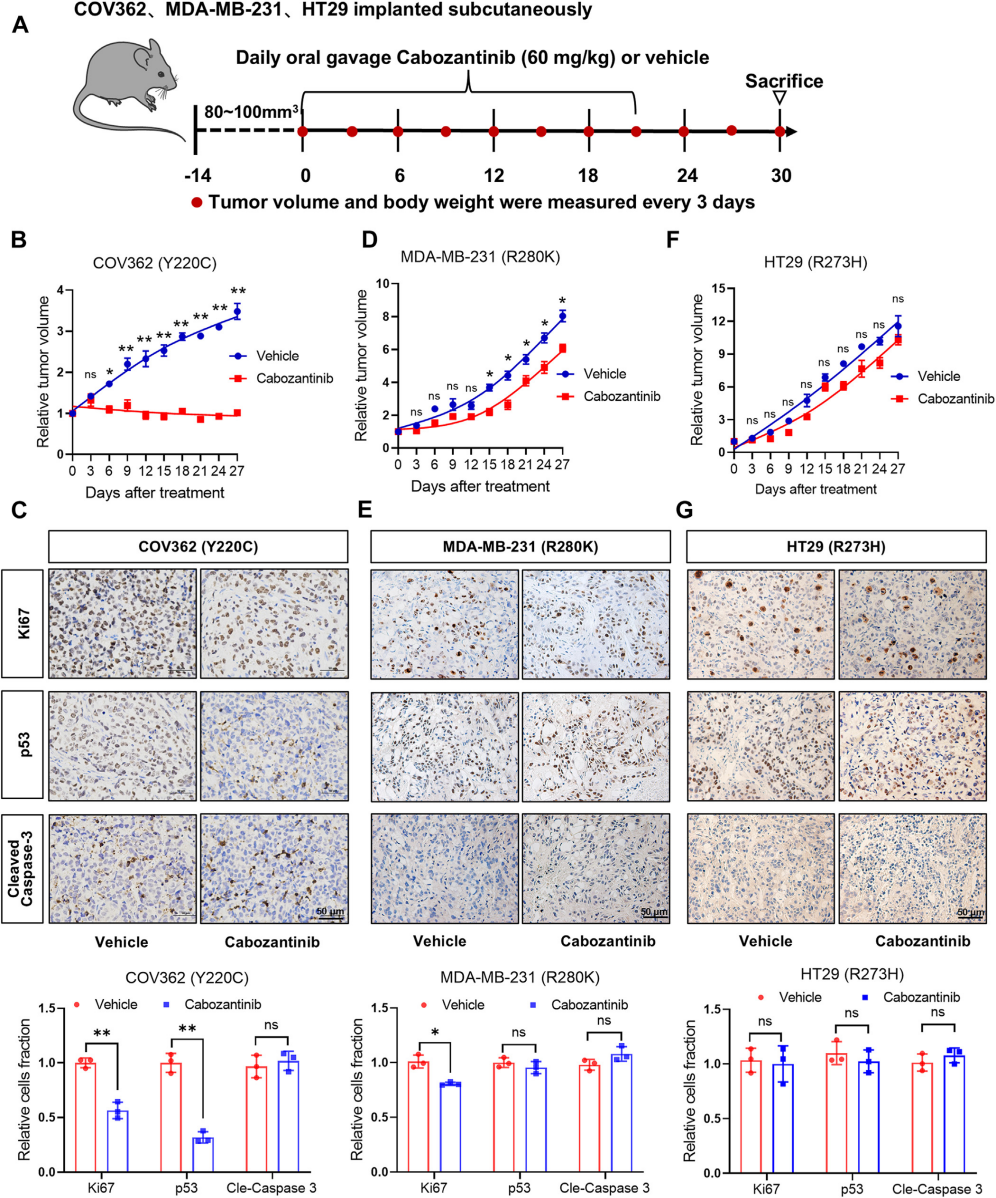
**Cabozantinib preferentially impedes p53**^**Y220C**^**-harboring tumor growth *in vivo*.***A*, Experimental regimen. *B–G*, The nude mice were inoculated with COV362, MDA-MB-231, and HT29 cells. Cabozantinib was administered to the mice *via* intraperitoneal injection. Tumor volume was measured at 3-days intervals. Tumor specimens were stained for Ki67 protein, p53 protein, and cleaved-caspase3 protein. Data shown are the mean ± SD (n = 5).

To recapitulate our *in vitro* observation in animal tumor models, we determined the effects of cabozantinib on tumor growth in mouse tumor xenograft models (Fig. 6*A* and Fig. S7*A*). Fig. 6*B* showed that administration of cabozantinib (60 mg/kg, once daily for 3 weeks. PO) significantly suppressed tumor growth of COV362 cells (p53^Y220C^). Immunohistochemistry (IHC) analyses showed that cabozantinib treatment led to a reduction in p53 protein in the tumors, accompanied by significant decreases in Ki67 staining. However, there was no change in cleaved caspase-3 staining. (Fig. 6*C*). In contrast, moderate or slight inhibition of tumor growth was observed in the mice bearing MDA-MB-231 (p53^R280K^), HT29 (p53^R273H^), or RKO (p53^WT^) tumors and treated with cabozantinib (Fig. 6, *D* and *F* and Fig. S7*B*). IHC analyses showed that cabozantinib treatment did not reduce p53 and cleaved caspase-3 protein levels in MDA-MB-231, HT29, and RKO tumors and that there was a slight decrease in Ki67 staining in these tumors (Fig. 6, *E* and *G* and Fig. S7*C*). Moreover, overexpression of USP7 protein in COV362(p53^Y220C^) cells counteracted the tumor growth inhibition caused by cabozantinib (Fig. S7, *D* and *E*) and reversed Ki67 and p53 staining in these tumors treated with cabozantinib and showed no effect on cleaved caspase-3 protein levels within the tumors (Fig. S7*F*). These observations suggest that the selective cytotoxic and chemosensitizing effect of cabozantinib on the cancer cells harboring p53^Y220C^ mutation are attributed to its ability to selectively induce degradation of this p53 mutant protein.

## Discussion

Currently, therapeutic agents that directly target degradation of p53^Y220C^protein, the most prevalent mutation in certain tumor types (8) and is associated with approximately 125,000 new cancer patients per year worldwide (9), are not available yet. Despite a previous study claiming that drugs capable of abrogating expression of mutant TP53 proteins or blocking their GOF activities would not provide substantial general therapeutic benefit in cancers expressing mutant TP53 (24), our experimental data reported here indicate that cabozantinib effectively inhibits tumor progression by eliminating mutant p53 protein. We demonstrate a novel role for cabozantinib in selectively inducing degradation of the p53-Y220C mutant (Fig. 1), and this role of cabozantinib may account for its preferential cytotoxicity and antitumor efficacy against tumors harboring p53^Y220C^ (Figs. 5 and 6 and Figs. S6 and S7). These results provide rationale for the use of cabozantinib in treatment of patients with cancers harboring p53-Y220C mutant.

We show that USP7, a deubiquitinase type involved in the stabilization and removal of lysine-linked ubiquitin from various substrate proteins, physically interacts with p53^Y220C^ protein (Fig. 4*A*) and facilitates the deubiquitination and stabilization of p53^Y220C^ protein (Fig. 4*D* and Fig. S5*A*), and cabozantinib can inhibit the interaction between USP7 and p53^Y220C^, resulting in decreased ubiquitination and amount of p53^Y220C^ protein (Fig. 4). Cluster analysis results from molecular dynamics simulations on free WT TP53 model, WT TP53–cabozantinib complex model, free Y220C TP53 model, and Y220C TP53–cabozantinib complex model revealed that only conformation A was present in the F113-C124 loop of the Y220C TP53–cabozantinib complex model where both cabozantinib and Y220C mutation were considered (Fig. 2*F*). These data suggest a potential impact of this configuration alteration on binding between p53^Y220C^ and USP7. Further studies are needed to elucidate the details of the molecular mechanism of action of cabozantinib.

The stability of mutant p53 is modulated by several E3 ubiquitin ligases including MDM2 and CHIP (25, 26). It is plausible that USP7 collaborates with E3 ubiquitin ligases to regulate the ubiquitination status of p53^Y220C^ protein. The evidence presented in this study highlights the crucial role of antagonistic actions between CHIP and USP7 in controlling the cabozantinib-induced proteasomal degradation of p53^Y220C^ protein. Firstly, USP7 prevents proteasomal degradation of p53^Y220C^ by deubiquitinating it. Conversely, CHIP acts as an E3 ligase for p53^Y220C^ to promote its degradation (Fig. 3, *C* and *D*). Additionally, knockdown of CHIP completely abolishes the downregulation of p53^Y220C^ caused by USP7 knockdown (Fig. S5, *D* and *E*). Secondly, knockdown of USP7 does not further decrease the cabozantinib-mediated degradation of p53^Y220C^ (Fig. 4*B*), whereas knockdown of CHIP significantly attenuates the effect of cabozantinib on p53^Y220C^ degradation (Fig. 3*C*). Thirdly, destruction of USP7–p53^Y220C^ interaction by cabozantinib accelerates ubiquitination and subsequent degradation mediated by CHIP (Fig. 4*F*). These results demonstrate the critical roles of both USP7 and CHIP in regulating the cabozantinib-induced alteration of p53^Y220C^ stability.

Targeting mutant p53 proteins through promoting their degradation is an attractive strategy for the development of anticancer drugs. For instance, suberoylanilide hydroxamic acid, an FDA-approved HDAC inhibitor, destabilizes mutant p53 by preventing HDAC6–Hsp90 interaction to facilitate MDM2- and CHIP-mediated proteasomal degradation (27). Statins, cholesterol-lowering drugs, deplete mutp53 through the mevalonate pathway–DNAJA1 axis (28). Furthermore, a small molecule activator of SIRT1 has been shown to lead to the deacetylation of p53 resulting in the depletion of mutant p53 protein level (29). Cabozantinib has been appreciated as an inhibitor of various tyrosine kinases including VEGFR2, MET, AXL, and RET. Here, we identify p53-Y220C mutant as a new target of this drug. Interestingly, we have discovered that cabozantinib specifically inhibits the growth of tumors harboring the p53-Y220 mutation without inducing apoptosis. Moreover, our findings suggest that cabozantinib, as an exogenous agent, can selectively induce a stress response in p53-Y220C mutant cells. This response involves a specific mechanism that simultaneously degrades P53 while suppressing tumor growth, thereby exerting an anticancer effect. However, further investigation is required to elucidate the precise underlying mechanisms. Given the presence of p53-Y220C mutant in tumors and its roles in cancer progression, our finding may have important implication, that is, utilizing cabozantinib to treat various cancers that carry p53-Y220C mutation.

## Experimental procedures

### Cell lines and culture

The human ovarian cancer cell lines COV362, SKOV3, and ES-2, the human pancreatic carcinoma cell line BxPC3, the human hepatocellular carcinoma cell line HUH7, the non-small cell lung cancer cell line H1299, the human breast carcinoma cell lines MCF-7 and MDA-MB-231, the human pulmonary carcinoma cell line A549, the human colorectal cancer cell lines HT29, RKO, and HCT116, and the human embryonic kidney HEK293T cells were purchased from m American Type Culture Collection. The identity of these cell lines was recently verified by STR analysis. COV362, SKOV3, BxPC3, and HT29 cell lines were cultured in RPMI 1640 medium supplemented with 10% heat-inactivated fetal bovine serum, 100 units/ml of penicillin, and 100 mg/ml of streptomycin. HUH7, A549, H1299, HCT116, RKO, MDA-MB-231, and HEK293T cell lines were cultured in Dulbecco’s modified Eagle’s medium supplemented with 10% heat-inactivated fetal bovine serum, 100 units/ml of penicillin, and 100 mg/ml of streptomycin. ES-2 cell line was cultured in McCoy's 5A medium supplemented with 10% heat-inactivated fetal bovine serum, 100 units/ml of penicillin, and 100 mg/ml of streptomycin. Cells were cultured at 37 °C in a humidified atmosphere of 20% O_2_/5% CO_2_. All cultures were monitored routinely and found to be free of contamination by *mycoplasma* or fungi, discarded after 3 months, and new lines propagated from frozen stocks.

### Reagents and antibodies

The following antibodies were used in immunoblotting, co-immunoprecipitation, immunofluorescence, or IHC: anti-p53 (Cell Signaling Technology, catalog number 2524S), anti-CHIP (Cell Signaling Technology, catalog number 2080S), anti-c-Met (Abcam, catalog number ab279587), anti-VEGFR2 (Cell Signaling Technology, catalog number 2479S), anti-AXL (Cell Signaling Technology, catalog number 8661S), anti-USP7 (ABclonal Technology, catalog number A3448), anti-MDM2 (Santa Cruz Biotechnology, catalog number sc-965), anti-Myc (Cell Signaling Technology, catalog number 2278p), anti-HA (Cell Signaling Technology, catalog number 5017), anti-Flag (Cell Signaling Technology, catalog number 14793), anti-GAPDH (Cell Signaling Technology, catalog number 5174), anti-Ki67 (Proteintech, catalog number 19972-1-AP), and anti-Cleaved Caspase-3 (Cell Signaling Technology, catalog number 9661S; Cell Signaling Technology, catalog number 14220S). Cabozantinib was purchased from YEASEN. Paclitaxel (PTX; NSC 125973) was purchased from selleck. 3-methyladenine (catalog number HY-19312), MG132 (catalog number HY-13259), and cycloheximide (CHX; catalog number HY-12320) were purchased from Med Chem Express.

### SiRNA and plasmid transfection

Double-strand siRNAs for CHIP, USP7, c-Met, VEGFR2, and MDM2 were purchased from GenePharma. In all experiments, nontarget siRNA (GE Healthcare Life Sciences) was used as a negative control. Flag-p53^WT^, Flag-p53^R175H^, Flag-p53^R273H^, Flag-p53^Y220C^, Flag-p53^Y220C^-K370R, Flag-p53^Y220C^-K372R, Flag-p53^Y220C^-K373R, Flag-p53^Y220C^-K381R, Flag-p53^Y220C^-K382R, Flag-p53^Y220C^-K386R, Flag-USP7, Myc-p53^Y220C^, Myc-USP7, Flag-USP7, Myc-CHIP, HA-Ub, HA-Ub-K48 (in which all the lysine residues, except K48, are mutated), and HA-Ub-K63 (in which all the lysine residues, except K63, are mutated) plasmids were purchased from Public Protein/Plasmid Library. Transfection of siRNA and plasmids were carried out using lipofectamine 6000 (Beyotime Biotechnology) according to the manufacturer's protocol.

### LC-MS/MS label-free quantitative proteomics and bioinformatics analyses

LC-MS/MS label-free quantitative proteomics and bioinformatics analysis were conducted. Briefly, COV362 cells were transfected with Flag-vector or Flag-p53^Y220C^ plasmids for 24 h. And the COV362 cells which transfected with Flag-p53^Y220C^ plasmids was treated with 1 μl/ml DMSO or 10 μM cabozantinib for 40 h and then treated with 10 μM MG132 for an additional 8 h. Whole cell lysate preparation and protein extraction were carried out by a standard protocol. Proteins were digested for 20 h at 37 °C with sequencing grade modified trypsin (Promega, V5111). The Ettan MDLC system (GE Healthcare) was applied for desalting and separation of tryptic peptide mixtures. The tryptic peptide mixtures were desalted through RP trap columns (Agilent Technologies, Zorbax 300, SB-C18) and separated on an RP column (Column Technology, 0.15 × 150 mm, RP-C18). The mobile phase was composed of 0.1% formic acid (A) and 0.1% formic acid in acetonitrile (B), with a gradient as follows: 0 to 105 min, 4 to 50% B; 105 to 114 min, 50 to 100% B; 114 to 120 min 100% B. The flow rate was kept at 2 μl/min. The MS analysis was performed on a Finnigan LTQ VELOS MS (Thermo Electron). Data-dependent MS/MS spectra were obtained simultaneously. Each scan cycle included one full MS1 scan in profile mode followed by 20 MS2 scans in centroid mode. Each sample was analyzed in triplicate. The nonredundant peptides were automatically searched according to the MS/MS spectra in the International Protein Index human protein database (version 3.53) using the TurboSEQUEST program in the Bioworks Browser software suite (version 3.1, Thermo Electron). Peptide quantification was based on the number of relevant peptide fragment MS/MS spectra in fully automatic mode using DeCyder MS Differential Analysis Software (version 2.0, GE Healthcare). The differentially expressed proteins were further analyzed according to the information from the GO database. Biological function classifications analyses were performed with the tools on GenMAPP (version2.1) database.

### Immunoblotting and co-immunoprecipitation

Proteins (10–20 μg) were resolved by SDS-PAGE and then transferred to PVDF membrane (Bio-Rad Laboratories). Membranes were incubated with primary antibodies (polyclonal antibodies were used for IP and monoclonal antibodies were used for immunoblotting) in 3% bovine serum albumin at 4 °C for overnight, followed by incubation with secondary antibodies at room temperature for 1 h. The protein signals were detected by ECL (Beyotime Biotechnology) method. For co-immunoprecipitation, appropriate antibodies were first incubated with protein A/G beads (Santa Cruz) at 4 °C for 6 h; cell lysates were followed by incubation with protein A/G beads or anti-Flag beads (sigma) at 4 °C for overnight. At the end of incubation, the beads were washed three times with RIPA lysis buffer, and the immunoprecipitates were eluted with a SDS buffer and then subjected to immunoblotting. Protein levels were quantified using ImageJ.

### Cell viability assay

Cellular viability was measured by 3-(4,5-dimethylthiazol-2-yl)-2,5-diphenyltetrazolium bromide assay. Briefly, cells were plated at a density of 5 × 10^3^ cells/well in 96-well tissue culture plates and subjected to different treatments. Following 48 h incubation at 37 °C in a humidified atmosphere containing 5% CO_2_, 95% air, the cells were incubated for another 4 h with 3-(4,5-dimethylthiazol-2-yl)-2,5-diphenyltetrazolium bromide reagent. The formazan product was dissolved in DMSO and read at 490 nm on a Victor3 Multi Label plate reader (PerkinElmer Life Sciences).

### Colony-formation assay

COV362 cell subjected to different treatments were plated in 35-mm tissue culture dishes. Following incubation at 37 °C in a humidified atmosphere containing 5% CO_2_/95% air for 14 days, cells were stained with 1% methylene blue in 50% methanol and colonies (>50 cells) were counted.

### Quantitative real-time PCR

Total RNA was prepared using Trizol reagent (Roche). First-strand complementary DNA was synthesized using Omniscript reverse transcription kit (Qiagen) with random primers. Quantitative reverse transcriptase–PCR was performed on ABI 7500 using Brilliant II SYBR Green QPCR master mix (Stratagene) and primers. After 40 cycles, data were collected and analyzed using the 7500 software (ABI).

### Organoid culture and drug sensitivity test

Lung cancer tissues identified as P53^Y220C^ mutations were obtained from patients. The organoids were isolated and cultured as described in references (30, 31, 32). Briefly, the patient-derived donor specimen (∼0.25–1 cm^3^) was minced and incubated at 37 °C with the Organoid Recovery Solution (bioGenousTME 238006). Incubation was left for 2 to 5 h to overnight according to the degree of liver fibrosis. After 2 to 5 h digestion, the digestion preparation was visually inspected and either digestion was stopped or, if a significant part of the original tissue was still under-digested (>50% of starting material, depending on the fibrotic status of the tissue), the preparation was left on 37 °C in the digestion solution. The digestion was stopped once no pieces of tissue were left, and the suspension was then filtered through a 100 μm nylon cell strainer and spun 3 min at 1500 rpm. The pellet was washed in Lung Adenocarcinoma Organoid Kit (bioGenousTM K2138-LA) and then mixed with Organoid Culture ECM (bioGenousTM M315066). 2000 to 5000 cells were seeded per well in a 24-multi-well plate. After Organoid Culture ECM had solidified, the organoids were passaged between 2 and 4 weeks after initial seeding. Organoids were enzymatically dissociated into single-cell suspensions and seeded into 384-well plates for drug testing. Organoid were treated with a fixed Cabozantinib concentration (2.5 μM) and a concentration series of PTX. Cell viability was assayed by Organoid Viability ATP Assay Kit (bioGenous TM E238003). CompuSyn software (www.combosyn.com) was used to determine the potential synergism of two drugs. Combination index values lower than 1.0 were considered synergistic.

### IHC analysis of tissue specimens

For IHC analysis of xenograft tumors, tumor specimens were fixed in 4% paraformaldehyde and stained with H&E. For *in situ* determination of cell proliferation, sample slides were incubated with anti-Ki67 antibody (1:200 dilution), anti-Cleaved Caspase-3 antibody (1:200 dilution). For *in situ* determination of p53 protein expression, sample slides were incubated with anti-p53 antibody (1:200 dilution).

### Immunofluorescence staining

For mitosis, cells were washed with PBS, fixed with pre-cold paraformaldehyde for 10 min, and then permeabilized with 0.1% Triton X-100 in PBS for 10 min at room temperature. Following incubation in the blocking buffer (5% BSA) for 1 h at room temperature, the samples were incubated with an anti-p53 monoclonal antibody (1:200 dilution) at 4 °C for overnight. At the end of incubation, the cells were washed three times in PBS and then stained with Alexa Fluor 594 Goat Anti-Mouse IgG (1:200 dilution, Invitrogen) for 1.5 h at room temperature. Nuclei were stained with DAPI (1:1000,sigma) for 30 min. Images were acquired on confocal microscope.

### Purification of GST fusion protein

Purification of p53^Y220C^: *Escherichia coli* (BL21) transformed with GST-p53^Y220C^ was incubated with 0.2 mM of IPTG for 4 h. The GST fusion proteins were purified from bacterial lysates with GSH-Sepharose 4B beads, following the manufacturer’s instructions (Amersham Biosciences Corp.); purification of USP7: *E. coli* transformed with His-Flag-USP7 was incubated with 0.2 mM of IPTG for 4 h. Supernatants containing the N-terminal His-tagged Flag-USP7 proteins were filtered through a 0.45-μm membrane, applied to a prepacked His-Trap excel Ni^2^+ metal affinity chromatography column. Bound USP7 proteins were eluted with a linear gradient of 18 column volumes of imidazole. Fractions containing USP7 proteins as determined by SDS-PAGE and were digested with HRV 3C Protease and dialyzed to 10 mM IMID overnight at 4 °C. The supernatant was loaded onto a HiTrap Q HP anion exchange chromatography column equilibrated in 20 mM Tris–HCl buffer, pH 8.0, 0.1 mM TCEP. USP7 proteins were eluted using a linear gradient of 13 column volumes of NaCl (0–1 M) in the same buffer. Fractions containing USP7 proteins were pooled, concentrated to about 100 mg/ml, and performed SEC in 20 mM Tris–HCl, pH 8.0, 150 mM NaCl, 1 mM TECP, 5% glycerol. All column chromatography purifications were performed on an AKTA Pure 25 system at 4 °C.

### GST pull-down assay

The plasmids for GST-p53 and Flag-USP7 were transfected into *E. coli*. The fusion proteins were prepared as described previously. Approximately 100 μg of GST-p53 fusion protein was immobilized in 50 μl of glutathione agarose and equilibrated before being incubated together at 4 °C for 60 min with gentle rocking motion. Approximately 100 μg of Flag-USP7 fusion protein was added to the immobilized GST-p53 after three washes with PBST. The two fusion proteins were incubated 24 h at 4 °C with DMSO or cabozantinib under gentle rotation. The bound proteins were eluted with elution buffer (10 mM glutathione in PBS, pH 8.0) and analyzed by immunoblotting.

### Generation of stable cell line

A human USP7 cDNA was inserted into the pLVX-AcGFP lentiviral vector (Clontech). To generate lentiviral particles, HEK293T cells at 80% confluence were transfected with 10 μg of pLVX-AcGFP-USP7, 3.5 μg of VSV-G envelope glycoprotein, 2.5 μg of packaging proteins (Rev), and 6.5 μg of packaging proteins (ΔR8.74) by using PEI (Sigma) as a gene delivery carrier. After being washed and refreshed with media, cells were further cultured for 48 h to produce lentivirus. The lentivirus particle-enriched supernatants were harvested, filtered, and stored frozen at −80 °C for further use. To generate cells stably overexpressing USP7, COV362 cells were transfected with 2 μg of pLVX-AcGFP-USP7 or pLVX-AcGFP-vector using Lipofectamine 2000 according to manufacturer’s instructions. Forty hours after transfection, the cells were selected positivity by Puromycin (1.5 μg/ml). Positive clones were selected for further investigation.

### Animal experiments

Animal maintenance and experimental procedures were approved by the Institutional Animal Care and Use Committee of Soochow University. Immunodeficiency NOD/SCID-based immunocompromised mice (5–6 weeks old, female, n = 5) were inoculated subcutaneously with COV362, HT29, MDA-MB-231, RKO, COV362^pLVX-vector^, and COV362^pLVX-USP7^ cells (5 × 10^6^ cells/mouse). Two weeks after inoculation, mice were randomly divided into two groups and treated with cabozantinib (60 mg/kg) (33) by Po, every day for 21 days. Tumor volume and body weight were measured every 3 days, respectively. Tumor volume was determined by measuring the length (L) and the width (W) of the tumors and calculating using the formula: V = L × W^2^/2. At the end of the experiment, the mice were euthanized, and tumors were surgically dissected. The tumor specimens were fixed in 4% paraformaldehyde for histopathologic examination. All animal experiments followed the Institutional Animal Care and Use Committee of Soochow University approved protocol: K113201819.

### Statistical analysis

Statistical analyses were performed using Microsoft Excel software and GraphPad Prism. The results are presented as mean ± SD from at least three independent experiments. The *p* values for comparisons between experimental groups were obtained by Student’s *t* test. All statistical tests were two-sided. ∗: *p* < 0.05; ∗∗: *p* < 0.01, ns.: not statistically significant.

## Data availability

All data have been provided in this manuscript. Additional details regarding data and protocol supporting the findings of this study are available from the corresponding author upon request.

## Supporting information

This article contains supporting information.

## Conflict of interest

The authors declare that they have no conflicts of interest with the contents of this article.
