# Supplementary material for: Cabozantinib selectively induces proteasomal degradation of p53 somatic mutant Y220C and impedes tumor growth
Source: J Biol Chem. 2025 Jan 8;301(2):108167. doi: 10.1016/j.jbc.2025.108167 (PMC11847077; doi:10.1016/j.jbc.2025.108167)
Supplement: Figures S1-S7 and Table S1 [file mmc1.docx]

**Supporting Information**

**Cabozantinib Selectively Induces Proteasomal Degradation of p53 Somatic Mutant Y220C and Impedes Tumor Growth**

**FangLin Lv^#,a^, Lu Zhang^#,a^,** **Cheng Ji^#,b^, Lei Peng^a^, Mingxian Zhu^a^, Shumin Yang^a^, Shunli Dong^a^, Mingxuan Zhou^a^, Fanfan Guo^a^, Zhenyun Li^a^, Fang Wang^c^, Youguo Chen^c^, Jinhua Zhou^c^, Xingcong Ren^c^, Genhai Shen^a^, Jin-Ming Yang^d^, Bin Li^a^*, Yi Zhang^a^***

^a^Department of Hepatopancreatobiliary Surgery, Suzhou Ninth Hospital Affiliated to Soochow University; Department of Pharmacology, College of Pharmaceutical Sciences, Soochow University; Jiangsu, China; ^b^Department of Respiratory Medicine, First Affiliated Hospital, Soochow University, Jiangsu, China; ^c^Department of Gynecology and obstetrics, First Affiliated Hospital, Soochow University, Jiangsu, China; ^d^Department of Cancer Biology and Toxicology, Markey Cancer Center, University of Kentucky, College of Medicine, Lexington, KY, 40506, USA;

# These authors contributed equally to this work.

*Address correspondence to: Bin Li, Department of Hepatopancreatobiliary Surgery, Suzhou Ninth Hospital Affiliated to Soochow University, 2666 Ludang Road, Jiangsu, China; E-mail address: bli4004@suda.edu.cn; Yi Zhang, Department of Pharmacology, College of Pharmaceutical Sciences, Soochow University, 199 Renai Road, Jiangsu, China, 215123; E-mail address: [zhangyi@suda.edu.cn](mailto:zhangyi@suda.edu.cn).

Includes-

1. Supporting Figure1-7
2. Supporting Table S1


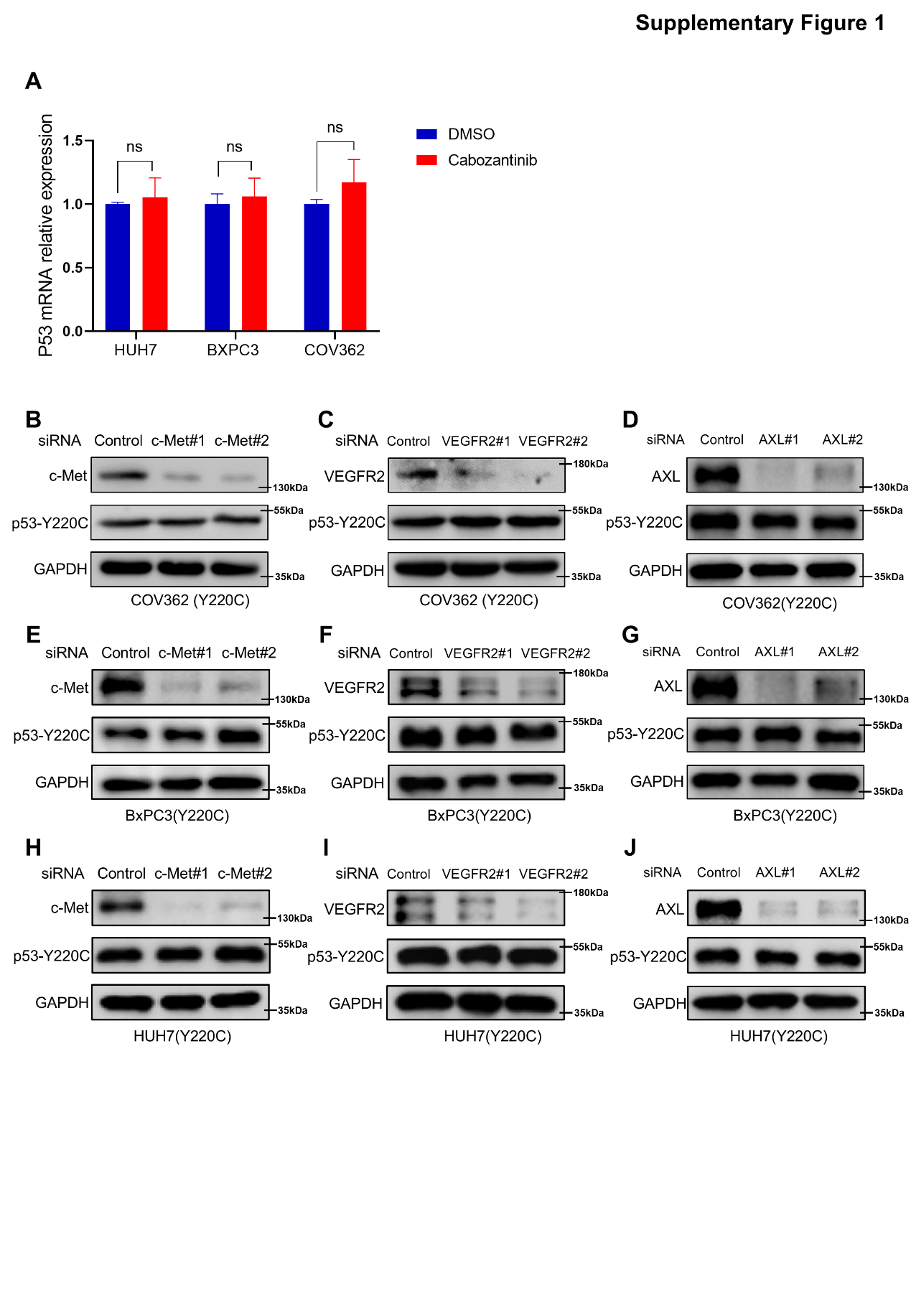


**Supporting Figure 1.** **Cabozantinib influence p53^Y220C^ through a unique mechanism.**

**(A)** Quantitative real-time PCR analysis of p53 RNA expression in COV362、 BxPC3 and HUH7 cells treated with DMSO or 10 μM cabozantinib for 48 hours . Statistical significance was determined by a two-tailed unpaired t-test. Data shown are the mean ± SD, (n = 4). **(B-J)** COV362, BxPC3 or HUH7 cells were treated with 10 μM cabozantinib for 48 hrs after transfection with the indicated siRNA for 24 hrs. c-Met, VEGFR2 or AXL and p53 were examined by immunoblotting. GAPDH was used as a loading control.


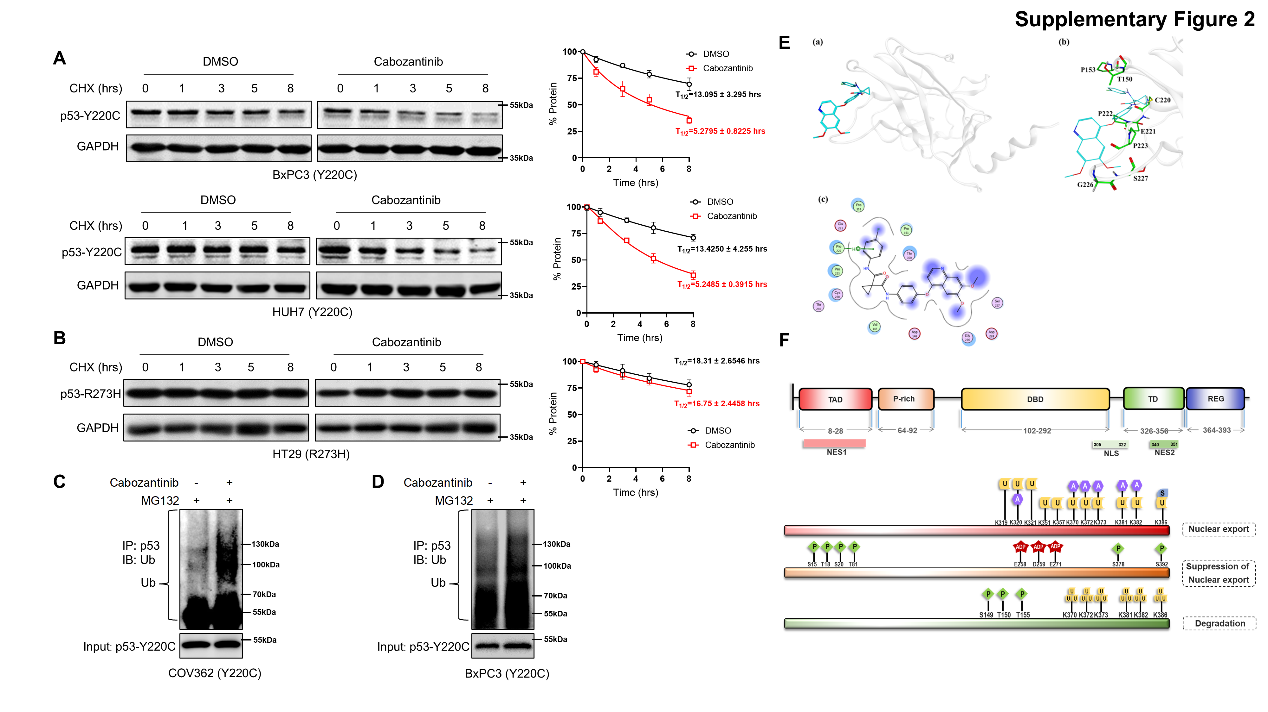


**Supporting Figure 2. Cabozantinib influence p53^Y220C^ through a unique mechanism and Cabozantinib alters the structure of p53-Y220C.**

**(A-B)** Immunoblotting for p53 in BxPC3 and HUH7 cells (A) and HT29 cells (B) were treated with DMSO or 10 μM cabozantinib for 48 hrs and then treated with 50 μg/ml cycloheximide (CHX) at different time points time before harvesting the cell for immunoblotting. Bars are mean ± SD (n =3). **(C-D)** COV362 (C) and BxPC3 (D) cells were treated with DMSO or 10 μM cabozantinib for 48 hr, and then 10 μM MG132 was added 8 hrs before harvesting the cells. Cell lysates were immunoprecipitated with an anti-p53 antibody. The immunoprecipitates and input were probed for Ub and p53 by immunoblotting. **(E)** **(a)** Overall view of binding mode between TP53 protein and ligand; **(b)** Detailed 3D binding mode between TP53 protein and ligand; **(c)** Detailed 2D binding mode between TP53 protein and ligand. The key residues of T150, C220, P222, G226 and S227 played key roles in the binding between TP53 protein and ligand. **(F)** Multiubiquitination modification site model of p53 protein.


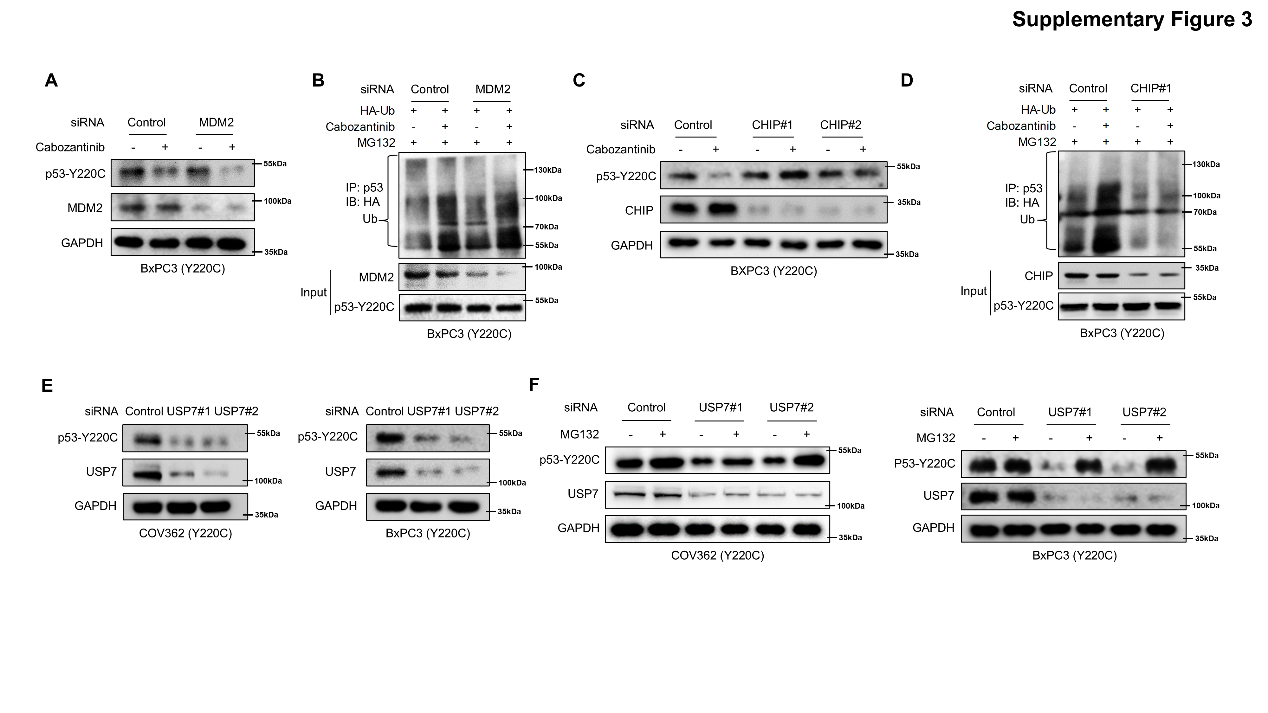


**Supporting Figure 3. Cabozantinib unleashes CHIP-mediated degradation by preventing USP7 interaction with p53-Y220C**

**(A)** BxPC3 cells were treated with 10 μM cabozantinib for 48 hrs after transfected with the indicated siRNA for 24 hrs. P53 and MDM2 were examined by immunoblotting. GAPDH was used as a loading control. **(B)** BxPC3 cells were treated with 10 μM cabozantinib for 48 hrs after transfected with the indicated siRNA for 24 hrs and then 10 μM MG132 was added 8 hrs before harvesting the cells. Cell lysates were immunoprecipitated with an anti-p53 antibody. The immunoprecipitates and input were probed for HA and p53 by immunoblotting. **(C)** BxPC3 cells were treated with 10 μM cabozantinib for 48 hrs after transfected with the indicated siRNA for 24 hrs. P53 and CHIP were examined by immunoblotting. GAPDH was used as a loading control. **(D)** BxPC3 cells were treated with 10 μM cabozantinib for 48 hrs after transfected with the indicated siRNA for 24 hrs and then 10 μM MG132 was added 8 hrs before harvesting the cells. Cell lysates were immunoprecipitated with an anti-p53 antibody. The immunoprecipitates and input were probed for HA and p53 by immunoblotting. **(E)** COV362 and BxPC3 cells were treated with 10 μM cabozantinib for 48 hrs after transfected with the indicated siRNA for 24 hrs. P53 and USP7 were examined by immunoblotting. GAPDH was used as a loading control. **(F)** COV362 and BxPC3 cells were transfected with the indicated siRNA for 24 hrs, either in the presence or absence of MG132 (10 µM) for 8 hrs before harvesting the cells. P53 and USP7 were examined by immunoblotting. GAPDH was used as a loading control.


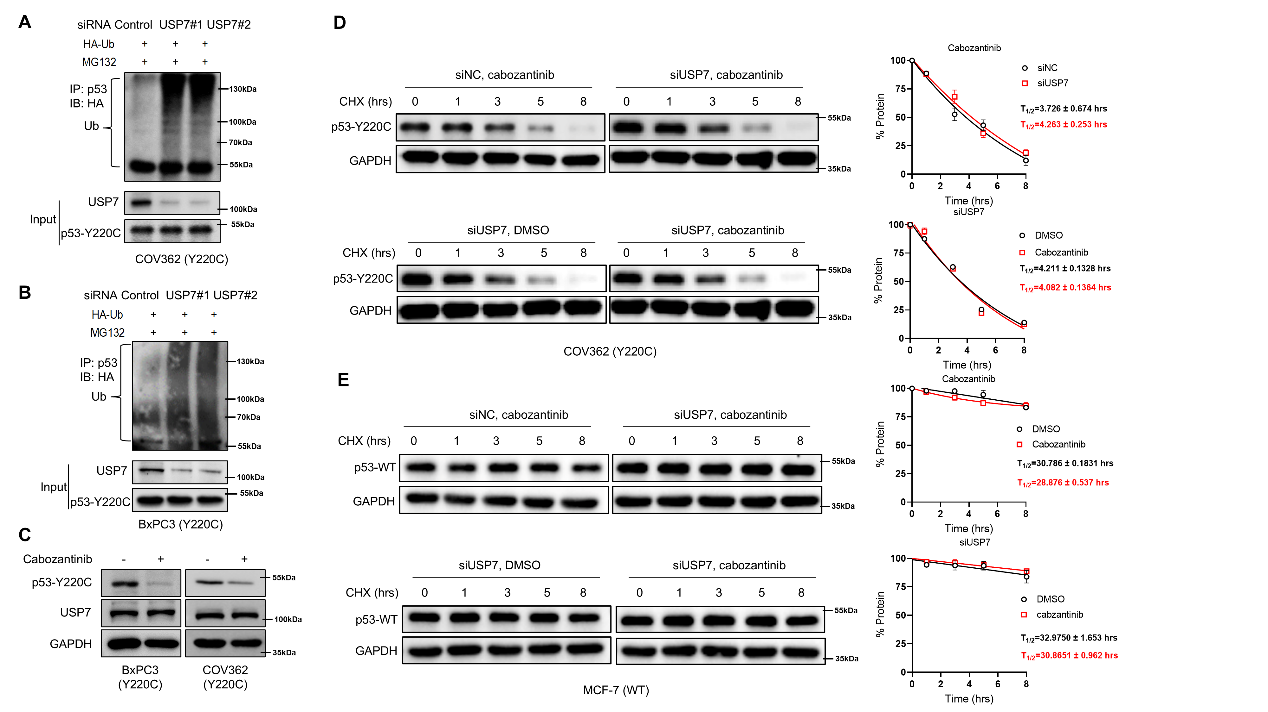


**Supporting Figure 4. USP7 played a key role in CHIP-mediated p53-Y220C degradation. (A-B)** COV362 and BxPC3 cells were treated with 10 μM cabozantinib for 48 hrs after transfected with the indicated siRNA for 24 hrs and then 10 μM MG132 was added 8 hrs before harvesting the cells. Cell lysates were immunoprecipitated with an anti-p53 antibody. The immunoprecipitates and input were probed for HA and p53 by immunoblotting. **(C)** Immunoblotting for p53 and USP7 in BxPC3 and COV362 cells treated with 10 μM cabozantinib for 48 hrs. GAPDH was used as a loading control. **(D-E)** COV362 (p53Y220C) and MCF-7 (p53WT) cells were transfected with the indicated siRNA for 24 hrs, and then treated with 10 μM cabozantinib for 48 hrs, either in the presence or absence of MG132 (10 µM) for 8 hrs before harvesting the cells. GAPDH was used as a loading control. Bars are mean ± SD (n = 3).


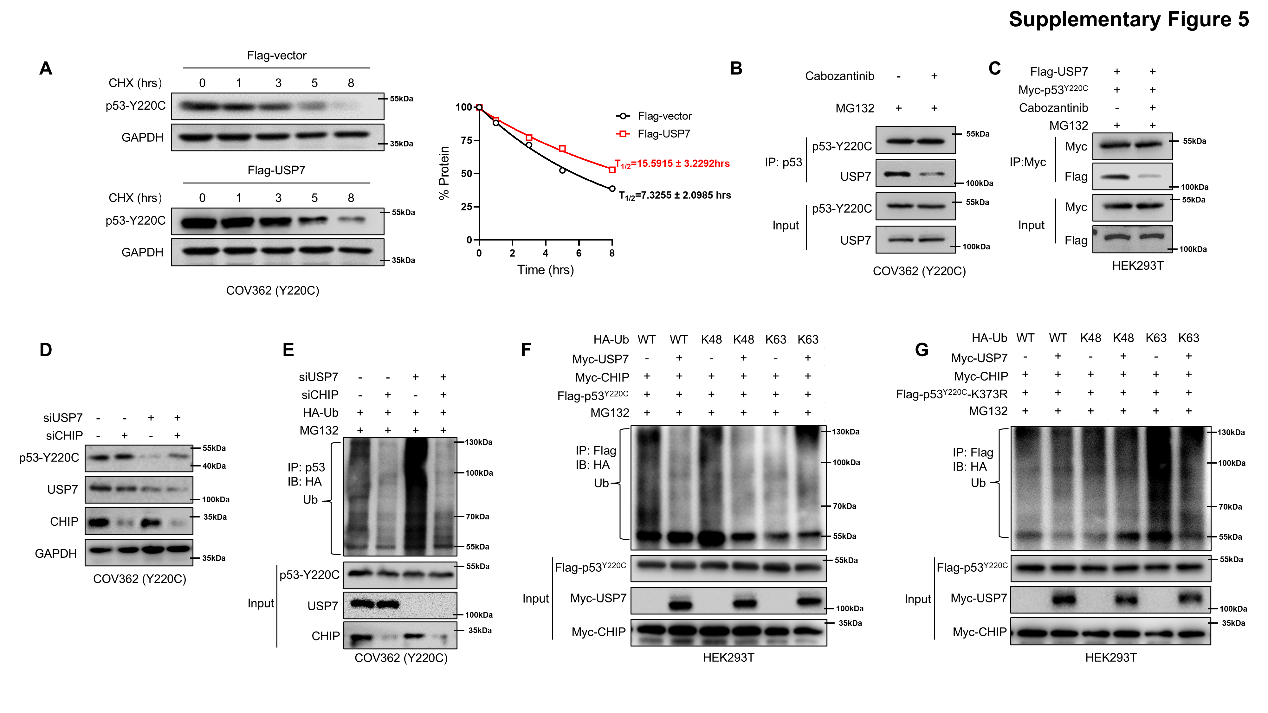


**Supporting Figure 5. USP7 played a key role in CHIP-mediated p53-Y220C degradation.**

**(A)** COV362 cells transfected with indicated Flag-vector plasmid or Flag-USP7 plasmid for 24 hrs were treated with DMSO or 10 μM cabozantinib for 48 hrs and then treated with 50 μg/ml cycloheximide (CHX) at different time points time before harvesting the cell for immunoblotting. Bars are mean ± SD (n = 3). (B) COV362 cells were treated or untreated with cabozantinib (10 μM) for 48 hrs and then 10 μM MG132 was added 8 hrs before harvesting the cells. Cell lysates were immunoprecipitated with an anti-p53 antibody. The immunoprecipitates and input were probed for p53 and USP7 by immunoblotting. **(C)** HEK293T cells were treated with 10 μM cabozantinib for 48 hrs after transfected with the indicated plasmids for 24 hrs and then 10 μM MG132 was added 8 hrs before harvesting the cells. Cell lysates were immunoprecipitated with an anti-Myc antibody. The immunoprecipitates and input were probed for Myc and Flag by immunoblotting. **(D)** COV362 cells were transfected with the indicated siRNA for 24 hrs. P53, USP7 and CHIP were examined by immunoblotting. GAPDH was used as a loading control. **(E)** COV362 cells were transfected with the indicated siRNA for 24 hrs and then 10 μM MG132 was added 8 hrs before harvesting the cells. Cell lysates were immunoprecipitated with an anti-p53 antibody. The immunoprecipitates and input were probed for HA, p53, USP7 and CHIP by immunoblotting. **(F-G)** HEK293T cells were transfected with the indicated plasmids for 24 hrs and then 10 μM MG132 was added 8 hrs before harvesting the cells. Cell lysates were immunoprecipitated with an anti-Flag antibody. The immunoprecipitates and input were probed for HA, Flag and Myc by immunoblotting.


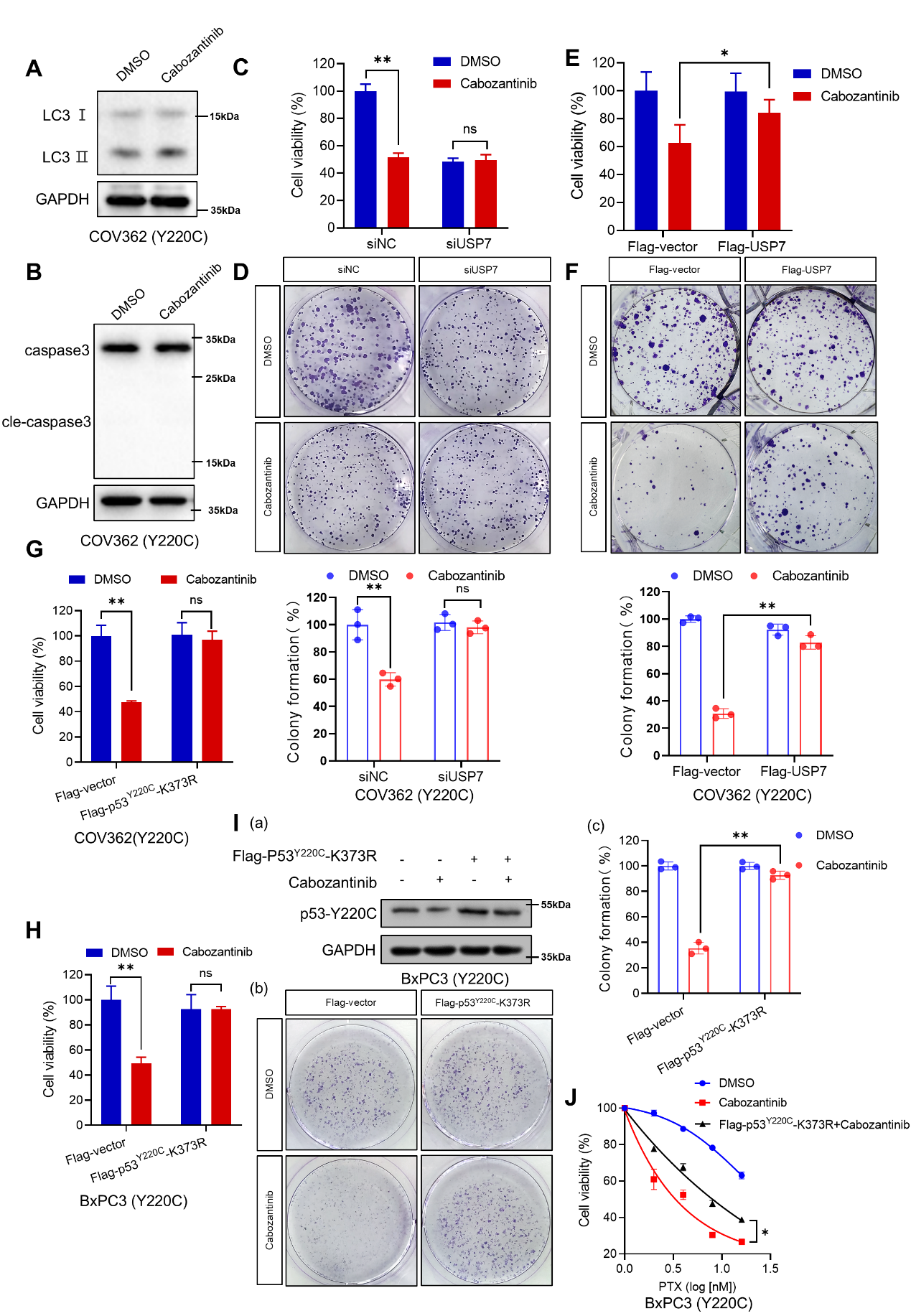


**Supporting Figure 6. The anti-tumor effect of cabozantinib and p53-Y220C degradation.**

**(A)** Immunoblotting of LC3 Ⅰ and LC3 Ⅱ in COV362 cells treated with 10 μM cabozantinib for 48 hrs. GAPDH was used as a loading control. **(B)** Immunoblotting of caspase 3 and cleaved caspase 3 in COV362 cells treated with 10 μM cabozantinib for 48 hrs. GAPDH was used as a loading control. **(C)** COV362 cells were transfected with siNC or siUSP7 for 24 hrs, followed by treatment with or without cabozantinib for 48 hrs. Cell viability was determined by MTT assay. Data shown are the mean ± SD (n = 6). **(D)** Colony formation assays for siNC or siUSP7 COV362 cells treated with 10 μM cabozantinib for 14 days. Data shown are the mean ± SD (n = 3). **(E)** COV362 cells were transfected with Flag-vector or Flag-USP7 plasmids for 24 hrs, followed by treatment with or without cabozantinib for 48 hrs. Cell viability was determined by MTT assay. Data shown are the mean ± SD (n = 6). **(F)** Colony formation assays for Flag-vector or Flag-USP7 COV362 cells treated with 10 μM cabozantinib for 14 days. Data shown are the mean ± SD (n = 3).  **(G)** COV362 cells were transfected with Flag-vector or Flag-p53^Y220C^-K373R plasmids for 24 hrs, followed by treatment with or without cabozantinib for 48 hrs. Cell viability was determined by MTT assay. Data shown are the mean ± SD (n = 6). **(H)** BxPC3 cells were transfected with Flag-vector or Flag-p53^Y220C^-K373R plasmids for 24 hrs, followed by treatment with or without cabozantinib for 48 hrs. Cell viability was determined by MTT assay. Data shown are the mean ± SD (n = 6). **(I)** (a) Flag-vector or Flag-p53^Y220C^-K373R BxPC3 cells were treated with or without cabozantinib for 48 hrs and then p53 protein was examined by immunoblotting. GAPDH was used as a loading control. (b-c) Colony formation assays for Flag-vector or Flag-p53^Y220C^-K373R BxPC3 cells upon 10 μM cabozantinib treatment for 14 days. Data shown are the mean ± SD (n = 3). **(J)** Cell viability assay for the BxPC3 cells were treated with the indicated concentrations of paclitaxel for 48 hrs in the presence or absence of cabozantinib (10 μM) or Flag-p53^Y220C^-K373R plasmid transfection. Data shown are the mean ± SD, (n = 3).


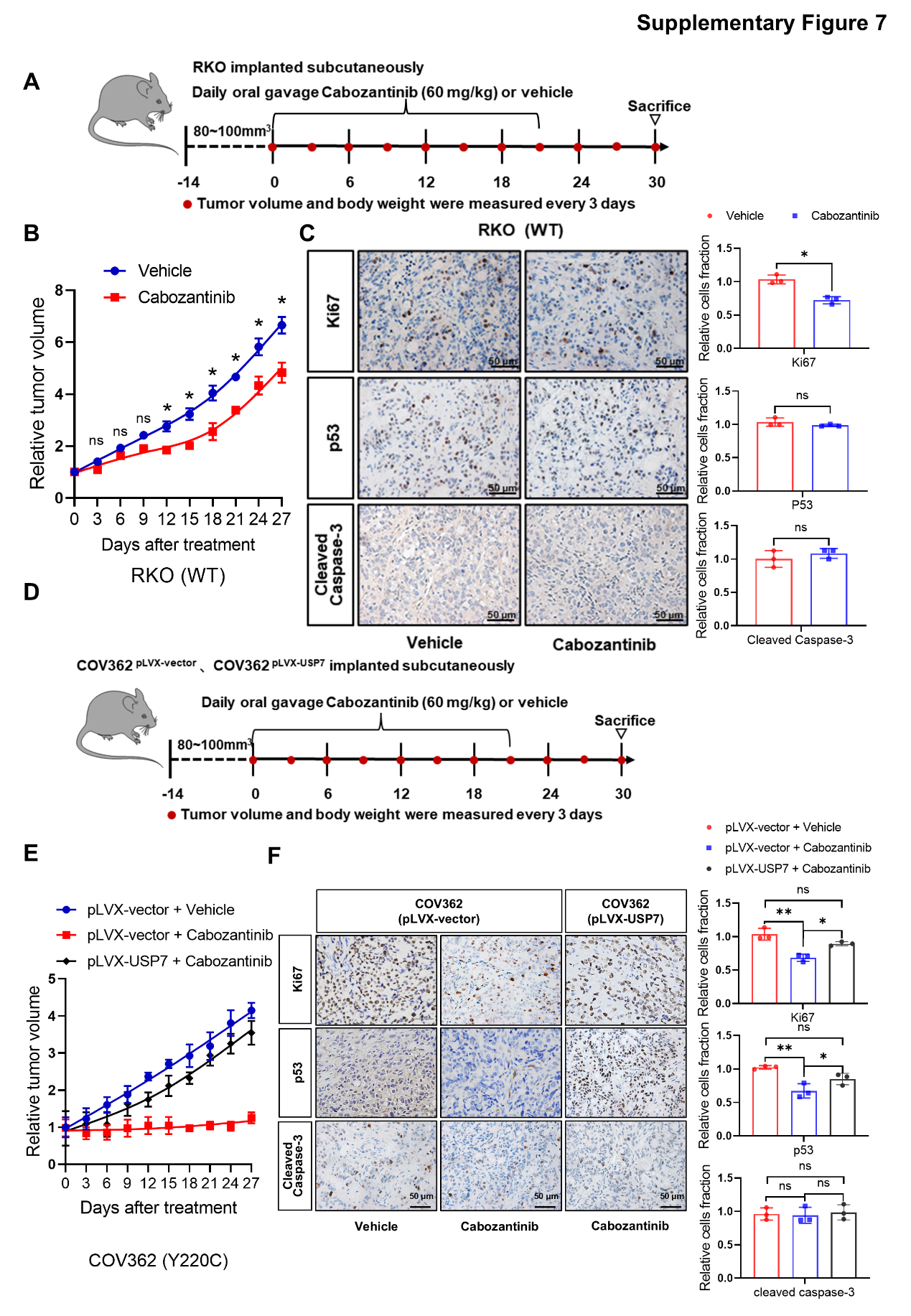


**Supporting Figure 7. Cabozantinib preferentially impedes p53^Y220C^-harboring tumor growth *in vivo*.** **(A)** Experimental regimen. **(B-C)** Nude mice were inoculated with RKO cells. Cabozantinib was administered to the mice via intraperitoneal injection. Tumor volume was measured at a 3-day interval. Tumor specimens were stained for Ki67 protein, p53 protein, and cleaved-caspase3 protein. Data shown are the mean ± SD, (n = 5). **(D)** Experimental regimen. **(E-F)** Nude mice were inoculated with COV362^pLVX-vector^ or COV362 ^pLVX-USP7^ cells. Cabozantinib was administered to the mice via intraperitoneal injection. Tumor volume was measured at a 3-day interval. Tumor specimens were stained for Ki67 protein, p53 protein, and cleaved-caspase3 protein. Data shown are the mean ± SD, (n = 5).

**Supporting Table S1.** The calculated IC50 of cell viability assay for the indicated cell lines upon cabozantinib treatment for 48 hrs.

| **Cell line** | **p53 status** | **IC50 (μM)** |
| --- | --- | --- |
| BxPC3 | Mutant Y220C | 9.015 ± 1.5 |
| HUH7 | Mutant Y220C | 9.266 ± 1.0 |
| COV362 | Mutant Y220C | 9.464 ± 1.3 |
| H1299 | Null | 32.74 ± 2.2 |
| A549 | Wild type | 33.98 ± 1.5 |
| MCF-7 | Wild type | 21.35 ± 1.7 |
| MDA-MB-231 | Mutant R280K | 24.95 ± 1.3 |
